# Nine phenylethanoid glycosides from *Magnolia officinalis* var. *biloba* fruits and their protective effects against free radical-induced oxidative damage

**DOI:** 10.1038/srep45342

**Published:** 2017-03-28

**Authors:** Lanlan Ge, Wenhui Zhang, Gao Zhou, Bingxin Ma, Qigui Mo, Yuxin Chen, Youwei Wang

**Affiliations:** 1 Institute of TCM and Natural Products, School of Pharmaceutical Sciences, Wuhan University, Wuhan 430071, P. R. China; 2 MOE Key Laboratory of Combinatorial Biosynthesis and Drug Discovery, Wuhan University, Wuhan 430072, P. R. China

## Abstract

To systematically study the chemical constituents in *Magnolia officinalis* var. *biloba* fruits, nine phenylethanoid glycosides were isolated by solvent extraction, silica gel, and preparative high-performance liquid chromatography (HPLC). Their structures were elucidated by 1D and 2D NMR analyses, including COSY, HMQC and HMBC correlations, and HPLC analysis of sugar residue. Nine phenylethanoid glycosides, namely, magnoloside I_a_ (**1**), magnoloside I_c_ (**2**), crassifolioside (**3**), magnoloside I_b_ (**4**), magnoloside III_a_ (**5**), magnoloside IV_a_ (**6**), magnoloside II_a_ (**7**), magnoloside II_b_ (**8**) and magnoloside V_a_ (**9**), were first isolated from the *n*-butanol fraction of *Magnolia officinalis* var. *biloba* fruits alcohol extract. Free radical scavenging activities of the nine phenylethanoid glycosides were assessed using the DPPH, ABTS, and superoxide anion radical scavenging assays. Simultaneously, protective effects of all compounds against free radical-induced oxidative damage were evaluated by two different kinds of mitochondrial damage model. The protective effects were assessed by mitochondrial swelling, the formations of malondialdehyde (MDA) and lipid hydroperoxide (LOOH), the activities of catalase (CAT), glutathione reductase (GR) and superoxide dismutase (SOD). All phenylethanoid glycosides showed significant protective effects.

Magnolia bark extracts are derived from *Magnolia officinalis* Rehd. et Wils. (*M. officinalis*) or *Magnolia officinalis* Rehd. et Wils. var. *biloba* Rehd. et Wils. (*M. officinalis* var. *biloba*). They have been used as traditional Chinese medicine (TCM) for treating abdominal distention, nausea, vomiting, dyspepsia, cough, and asthma^1^. Modern pharmacological research showed that magnolia bark extracts have effective antibacterial^2^, antioxidant^3^,^4^, anti-inflammatory^5^,^6^, anti-anxiety^7^, anti-gastric ulcer^8^, antitumor^9^, neuroprotective^10^, and cardiovascular protection^11^ activities. The bioactive components of magnolia bark extracts include mainly neolignans, lignans, sesquiterpenes, alkaloids, and phenylethanoid glycosides^12^,^13^,^14^,^15^,^16^. Recently, magnolia bark extracts have been recognized as a food additive worldwide.

In 2002, the Ministry of Health of the People’s Republic of China passed the notice to further standardize the management of health food raw materials, including *M. officinalis* and *M. officinalis* var. *biloba* barks, into healthy food materials^17^. In 2008, magnolia bark extracts achieved GRAS status in the United States and were allowed to be added as an ingredient to chewing gums and mints sold in the United States; In 2009, magnolia bark extracts were approved as a new ingredient of food resource by the United Kingdom and European Union; In 2010, the United Kingdom authorities officially approved and specified that the maximum addition amount of magnolia bark extracts in gum and mints is 0.2%^18^,^19^. Michael *et al*. also confirmed that compressed mints and chewing gum containing bark extracts are effective against bacteria responsible for oral malodor^20^. These policies marked the transformation of magnolia bark extracts from TCM to food and dramatically expanded their application. China is the only country to cultivate *M. officinalis* and *M. officinalis* var. *biloba* (the plant area of *M. officinalis* var. *biloba* is wider than that of *M. officinalis*)^21^, which are obtained largely by cutting down trees and peeling off their barks. However, this destructive utilization pattern is unsuitable for ecological and environmental protection. Therefore, a sustainable utilization pattern must be established to change this situation. *M. officinalis* and *M. officinalis* var. *biloba* fruits, which are nutritious reproductive organs of these plants, can be harvested every year. However, whether their fruits can be used as an alternative food resource remains unknown.

We had already reported that phenylethanoid glycosides were isolated from *M. officinalis* var. *biloba* fruits in 2015^22^. Phenylethanoid glycosides, which exist mostly in Orobanchaceae plants^23^,^24^, have been demonstrated to possess diverse biological activities, such as antioxidant, anti-inflammatory, antibacterial, antiviral, antitumor, neuroprotective, and immunomodulatory effects^25^,^26^,^27^,^28^,^29^,^30^,^31^. However, whether the phenylethanoid glycosides isolated from *M. officinalis* var. *biloba* fruits have the same effects as those isolated from Orobanchaceae plants needs to be elucidated. Moreover, we have not validated whether the analogous components from their fruits can be associated with the traditional functions of magnolia bark extracts. Therefore, more studies are needed to further understand the function of phenylethanoid glycosides.

In view of the more abundant resources of *M. officinalis* var. *biloba*, we focused on the *n*-butanol fraction of the alcohol extract from *M. officinalis* var. *biloba* fruits and isolated nine phenylethanoid glycosides (Fig. 1). The isolation and structural elucidation of these nine phenylethanoid glycosides, as well as their free radical scavenging activities, are reported in this paper. Subsequently, their protective effects against free radical-induced oxidative damage in two different kinds of mitochondrial damage model were also evaluated.

## Results and Disscussion

### Structural identification of phenylethanoid glycosides

Compounds **1**–**9** were identified as phenylethanoid glycosides. Their structures were characterized by the phenylpropionyl group and benzene ethanol group through ester linkage and glycoside bond connected to the central sugar, respectively.

Compound **2** was isolated as a light yellow amorphous powder. Its molecular formula was C_29_H_36_O_15_, which was deduced from the molecular ion peak at *m/z* 623.1999[M-H]^−^ (calcd. C_29_H_35_O_15_, 623.1981) by HR-ESI-MS and supported by the ^13^C NMR spectral data. The IR spectrum of **2** displaced the characteristic absorption bands for the hydroxyl group (3410 cm^−1^), conjugated carbonyl group (1687 cm^−1^), aromatic rings (1604 and 1516 cm^−1^), and glycosidic group (816 cm^−1^). The UV spectrum obtained the maximum absorption at 206, 291, and 330 nm.

**Figure 2:**
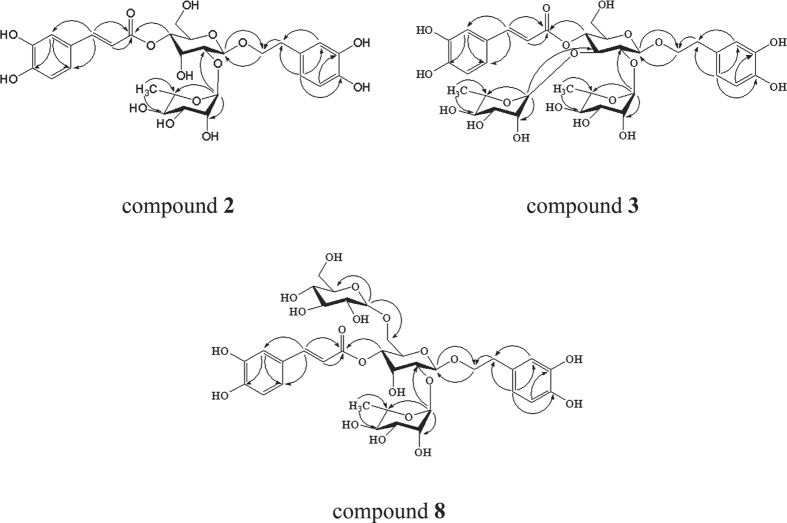
Key HMBC correlations (H → C) of compounds 2, 8, and 3.

The ^1^H-NMR and ^13^C NMR spectra of **2** (Tables 1, 2 and supplementary information) exhibited characteristic signals arising from the trans-caffeoyl group and 3,4-dihydroxy phenethyl alcohol group showing two sets of ABX-type signals caused by aromatic protons at *δ*_H_ 7.08 (1H, d, *J* = 2.0 Hz), 6.97 (1H, dd, *J* = 2.0, 8.0 Hz), 6.80 (1H, d, *J* = 8.0 Hz) and *δ*_H_ 6.73 (1H, d, *J* = 2.0 Hz), 6.71 (1H, d, *J* = 8.0 Hz), 6.58 (1H, dd, *J* = 2.0, 8.0 Hz), a pair of trans-olefinic proton signals at *δ*_H_ 7.63 (1H, d, *J* = 15.9 Hz), 6.33 (1H, d, *J* = 15.9 Hz), and benzylic methylene proton signal at *δ*_H_ 2.80 (2 H, t, *J* = 7.4 Hz), which were assigned to *δ*_C_ 115.30 (C-2′), 123.20 (C-6′), 116.45 (C-5′), 117.25 (C-2), 116.59 (C-5), 121.41 (C-6), 147.72 (C-7′), 114.71 (C-8′), and 36.73 (C-7), respectively, via HMQC correlation. Additionally, two anomeric proton resonances appeared at *δ*_H_ 4.85 (1H, d, *J* = 8.0 Hz) and 4.90 (1H, s), which correlated with signals at *δ*_C_ 100.47 (C-1″) and 97.91 (C-1″′) in the HMQC spectrum, respectively. The ^1^H-NMR spectrum also showed the presence of a methyl group at *δ*_H_ 1.29 (3H, d, *J* = 6.0 Hz), which indicated the presence of rhamnose in the compound. A series of signals in ^1^H-NMR (CD_3_OD, 400 MHz) at *δ*_H_ 4.85 (1H, d, *J* = 8.0 Hz), 3.47 (1H, m), 4.51 (1H, s), 4.84 (1H, m), 3.60 (1H, m), 3.62 (1H, m), and 3.79 (1H, m) and ^13^C NMR (CD_3_OD, 100 MHz) at *δ*_C_ 100.47 (C-1″), 73.19 (C-2″), 66.45 (C-3″), 70.57 (C-4″), 74.53 (C-5″), and 62.37 (C-6″) indicated a rare β-allopyranosyl unit. Moreover, this unit was confirmed by comparing the NMR features of those of magnolosides A, B, C, D, and E^16^,^32^,^33^ isolated from *M. officinalis* barks or *M. obovata* barks, which also contained allopyranose. Acid hydrolysate of compound **2** contained monosaccharide components, which were identified as D-allose and L-rhamnose by HPLC analysis. In the HMBC relationship of **2** (Fig. 2), the correlations between H-1″ (*δ*_H_ 4.85) and C-8 (72.30) showed that the phenylethanol moiety was linked to C-1″ (All-C-1″); the cross peak between H-4″ (*δ*_H_ 4.84) and C-9′ (168.14) showed that the trans-caffeoyl moiety was linked to C-4″ (All-C-4″); and the long-range correlations between H-1″′ (*δ*_H_ 4.90) and C-2″ (73.19) (All-C-2″) showed that the rhamnose moiety was linked to C-2″ (All-C-2″).

In light of all the above considerations, the structure of **2** was revealed as 2-(3, 4-dihydroxyphenyl)-ethyl-O-a-L-rhamnopyranosyl-(1 → 2)-(4-O-trans-caffeoyl)-β-D-allopyranoside.

Compound **3** was obtained as a canary yellow amorphous powder. The HR-ESI-MS data showed an accurate [M-H]^−^ ion at *m/z* 769.2668 (calcd. for C_35_H_45_O_19_, 769.2654), in accordance with the empirical molecular formula of C_35_H_46_O_19_. The IR spectrum of **3** displaced characteristic absorption bands for the hydroxyl group (3402 cm^−1^), conjugated carbonyl group (1699 cm^−1^), aromatic rings (1603 and 1517 cm^−1^), and glycosidic group (814 cm^−1^). The UV spectrum obtained the maximum absorption at 206, 280, and 330 nm.

The ^1^H-NMR and ^13^C NMR spectra of **3** (Tables 1, 2 and supplementary information) exhibited the same characteristic signals to prove the existence of the trans-caffeoyl group and 3,4-dihydroxy phenethyl alcohol group as compound **2**. By contrast, three sugar anomeric protons were observed at *δ*_H_ 5.01 (1H, s), 4.96 (1H, s), and 4.51 (1H, d, *J* = 7.7 Hz) and resonated at *δ*_C_ 102.32 (C-1″″), 103.07 (C-1″′), and 102.46 (C-1′) in ^13^C NMR, respectively. They also could be supported in the HMQC spectrum. Two methyl groups at *δ*_H_ 1.14 (3H, d, *J* = 6.0 Hz) and 1.29 (3H, d, *J* = 6.0 Hz), as well as *δ*_C_ 17.91 (C-6″′) and 17.69 (C-6″″), indicated that compound **3** may contain two rhamnoses. A series of signals in ^1^H-NMR (CD_3_OD, 400 MHz) at *δ*_H_ 4.51 (1H, d, *J* = 7.7 Hz), 3.97 (1H, m), 3.57 (1H, m), 4.96 (1H, m), 3.51 (1H, m), 3.52 (1H, m), and 3.63 (1H, m) and in ^13^C NMR (CD_3_OD, 100 MHz) at *δ*_C_ 102.46 (C-1″), 82.68 (C-2″), 80.43 (C-3″), 69.83 (C-4″), 75.50 (C-5″), and 61.88 (C-6″) showed that the central sugar was β-glucose. The acid hydrolysate of compound **3** further confirmed the existence of D-glucose and L-rhamnose, which were identified by HPLC analysis. In the HMBC relationship of **3** (Fig. 2), the correlations between H-1″ (*δ*_H_ 4.51) and C-8 (*δ*_C_ 71.86) showed that the phenylethanol moiety was linked to C-1″ (Glc-C-1″); the cross peak between H-4″ (*δ*_H_ 4.96) and C-9′ (*δ*_C_ 167.91) showed that the trans-caffeoyl moiety was linked to C-4″ (Glc-C-4″); and the long-range correlations between H-1″′ (*δ*_H_ 4.96) to C-2″ (*δ*_C_ 82.68) (Glc-C-2″) and H-1″″ (*δ*_H_ 5.01) to C-3″ (*δ*_C_ 80.43) (Glc-C-3″) unambiguously showed that the two rhamnose moieties were linked at C-2″ and C-3″, respectively.

All these results confirmed the structure of compound **3** as 2-(3, 4-dihydroxyphenyl)-ethyl-O-a-L-rhamnopyranosyl-(1 → 2)-[a-L-rhamnopyranosyl-(1 → 3)]-(4-O-trans-caffeoyl)-β-D-glucopyranoside, named crassifolioside^34^.

Compound **8** was obtained as a light yellow amorphous powder. The negative-ion quasi-molecular ion peak in its HRESI-MS data at *m/z* 785.2520 [M-H]^−^ (calcd. for C_35_H_45_O_20_, 785.2510) together with the ^13^C NMR spectral data suggested a molecular formula of C_35_H_46_O_20_. The IR spectrum of **8** displaced characteristic absorption bands for the hydroxyl group (3234 cm^−1^), conjugated carbonyl group (1692 cm^−1^), aromatic rings (1604 and 1518 cm^−1^), and glycosidic group (813 cm^−1^). The UV spectrum obtained the maximum absorption at 205, 280, and 330 nm.

Comparison of the ^1^H-NMR (CD_3_OD, 400 MHz) and ^13^C NMR (CD_3_OD, 100 MHz) spectra of **8** (Tables 1, 2 and supplementary information) with those of **2** suggested that compound **8** contained one more sugar group than **2**. The extra series of sugar signals in ^1^H-NMR at *δ*_H_ 4.30 (1H, d, *J* = 7.7 Hz), 3.60 (1H, m), 3.25 (1H, m), 3.32 (1H, m), 3.35 (1H, m), 3.64 (1H, m), and 3.86 (1H, m) and in ^13^C NMR at *δ*_C_ 104.92 (C-1″″), 74.41 (C-2″″), 77.78 (C-3″″), 71.48 (C-4″″), 77.90 (C-5″″), and 62.67 (C-6″″) deduced that the extra sugar group was β-glucose. Acid hydrolysis of **8** further gave D-allose, D-glucose, and L-rhamnose as sugar residues by HPLC analysis. The 7.25 ppm down-field shift of C-6″ in the ^13^C NMR spectrum indicated the attachment of the extra sugar group to C-6″ of the central sugar. This finding was also supported by the HMBC correlation (Fig. 2) of H-1″″ (*δ*_H_ 4.30) and C-6″ (*δ*_C_ 69.62).

Based on the above evidence, compound **8** was established as 2-(3, 4-dihydroxyphenyl)-ethyl-O-a-L-rhamnopyranosyl-(1 → 2)-[β-D-glucopyranosyl-(1 → 6)]-(4-O-trans-caffeoyl)-β-D-allopyranoside.

**Figure 3:**
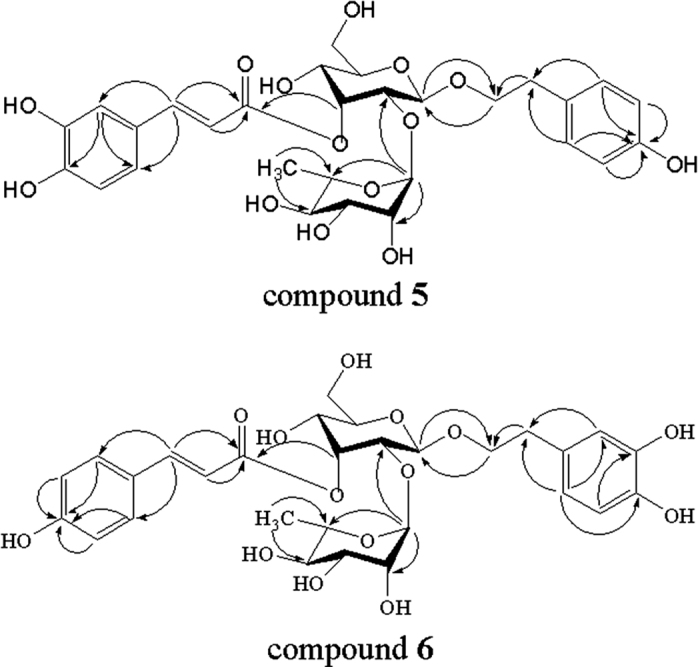
Key HMBC correlations (H → C) of compounds 5 and 6.

Compounds **5** and **6** were isolated as yellow amorphous powder, with the molecular formula of C_29_H_36_O_14_, as deduced from the [M-H]^−^ peak at *m/z* 607.2011 (calcd. for C_29_H_35_O_14_, 607.2021) and 607.2009 (calcd. for C_29_H_35_O_14_, 607.2021) via HR-ESI-MS and supported by the ^13^C NMR spectral data. The ^1^H-NMR and ^13^C NMR spectra of **5** (Table 3 and supplementary information) were very similar to those of compound **1** (magnoloside A), except for the signals caused by the 4-hydroxyphenylethyl alcohol group {*ortho*-coupled A_2_B_2_-type aromatic protons [*δ*_H_ 7.10 (2 H, d, *J* = 2.0 Hz), 6.71 (2 H, d, *J* = 8.0 Hz)] and two methylenes [*δ*_H_ 2.85 (2 H, t, *J* = 8.0 Hz), 3.67 (2 H, m)]}, which was also supported by the literature^35^. Comparison of the NMR data of **6** (Table 3 and supplementary information) with those of compound **1** (magnoloside A) revealed that the caffeoyl group of magnoloside A was replaced by the *trans*-p-coumaroyl group {*ortho*-coupled A_2_B_2_-type aromatic protons [*δ*_H_ 7.51 (2 H, d, *J* = 8.0 Hz), 6.84 (2 H, d, *J* = 8.0 Hz)] and *trans*-olefinic protons [7.68 (1H, d, *J* = 15.9 Hz), 6.45 (1H, d, *J* = 15.9 Hz)]}, which was also supported by the literature^25^. Furthermore, the HMBC correlation of **5** and **6** (Fig. 3) also showed similar modes of long-range correlations with **1**. Therefore, the structures of compounds **5** and **6** were elucidated as 2-(4-hydroxyphenyl)-ethyl-O-a-L-rhamnopyranosyl-(1 → 2)-(3-O-trans-caffeoyl)-β-D-allopyranoside and 2-(3, 4-dihydroxyphenyl)-ethyl-O-a-L-rhamnopyranosyl-(1 → 2)-(3-O-trans-coumaroyl)-β-D-allopyranoside, respectively.

The other compounds were identified as magnoloside A (**1**), magnoloside B (**7**), magnoloside D (**4**), and magnoloside E (**9**) by comparing their ^1^H-NMR and ^13^C NMR data with those reported in the literature^16^. All compounds were obtained from *M. officinalis* var. *biloba* fruits for the first time.

In the last few years, some confusing nomenclatures were found in the original articles^36^,^37^. For example, compound **2** and compound **8,** mentioned in this article, were both given the same nomenclature as magnoloside F though they actually posses different structures, while magnoloside F^36^ and magnoloside M^37^ were characterized by the same structure (compound **2**, mentioned in this article) but different nomenclature. In this manuscript, we propose a reasonable rule of nomenclature in view of the rich structure type of phenylethanoid glycosides. Thus, the confusion and ambiguity caused by two research groups could be clarified. Different types of phenylethanoid glycosides are numberd with Roman numerals (I, II, III, IV, V…), and isomers with subscripts a, b, c… are distinguished. Therefore, compounds **1, 2, 4, 5, 6, 7, 8** and **9** were renamed magnoloside I_a_ (old name was magnoloside A^32^), magnoloside I_c_ (old names were magnoloside F^31^ and magnoloside M^37^), magnoloside I_b_ (old name was magnoloside D^16^), magnoloside III_a_ (old name was magnoloside H^36^), magnoloside IV_a_ (old name was magnoloside G^36^), magnoloside II_a_ (old name was magnoloside B^33^), magnoloside II_b_ (old name was magnoloside F^37^), magnoloside V_a_ (old name was magnoloside E^16^).

### Free radical scavenging activities and their structure–activity relationship

*In vitro* DPPH radical scavenging, ABTS radical scavenging, and superoxide anion radical scavenging activities of the isolated phenylethanoid glycosides are summarized in Table 4. The table illustrates that all the isolated phenylethanoid glycosides showed excellent free radical scavenging activity. The analysis of the structure–activity relationship of these phenylethanoid glycosides in the free radical scavenging activity assay suggested that the presence of two adjacent phenolic groups in the molecule resulted in strong free radical scavenging activity. The more two adjacent phenolic groups were, the stronger the free radical scavenging activity was. Meanwhile, all isolated phenylethanoid glycosides were cinnamic acid derivatives, which contain α,β-conjugated unsaturated ester structures, thereby increasing benzene ring plane conjugation and allowing electron delocalization to stabilize free radicals. This conclusion was well supported by previous reports^38^,^39^.

In the DPPH radical scavenging assay, compound **1** and its structural analogs (**2**, **4**, and **9**) showed significant DPPH radical scavenging activity with far smaller IC_50_ values (11.79 ± 0.57, 12.99 ± 0.48, 16.23 ± 0.16, and 20.99 ± 0.50 μM, respectively) than the positive controls, such as V_C_ (40.94 ± 0.78 μM) and BHT (89.94 ± 4.57 μM). Compounds **3**, **7**, and **8** expressed inferior activity compared with the above four compounds because their IC_50_ values (21.38 ± 0.52, 22.94 ± 0.26, and 24.62 ± 0.15 μM, respectively) were near the value of V_C_. Moreover, Compound **5** and its structural isomer **6** showed large IC_50_ values (32.18 ± 0.97 and 35.17 ± 0.22 μM, respectively). Some observations could be made according to the above results. Compounds **3**, **7**, and **8** possessed larger steric hindrance because they contained three sugars, whereas compounds **1**, **2**, **4**, and **9** only contained two sugars. The increased steric hindrance effect of compounds **3**, **7**, and **8** prevented them from easily approaching the free radicals, so their DPPH radical scavenging capacity was relatively weak than compounds **1**, **2**, **4**, and **9**. Moreover, compared with the other seven compounds, compounds **5** and **6** belonged to phenylethanoid glycosides with two adjacent phenolic groups only in one side, so they exhibited poor activity.

The ABTS radical scavenging assay demonstrated that compounds **1, 2**, and **4** also exhibited good ABTS radical scavenging activity, but the IC_50_ value (6.23 ± 0.06 μM) of compound **9** was large. This result was different from that of the DPPH radical scavenging assay, indicating that the apiose group in compound **9** produced a negative effect on the ABTS radical scavenging assay. By contrast, the ABTS radical scavenging ability of compound **3** was far better than that of compounds **7** and **8**, which could be due to the glucose group in compound **3**. The superoxide anion radical scavenging assay showed that the activity of compound **9** was the best, whereas the activity of compound **3** was the worst. These completely opposite results may be due to the fact that the apiose group in compound **9** enhanced the influence on the superoxide anion radical scavenging assay, whereas the glucose group in compound **3** minimized this influence. These different results obtained from three assays may be explained by the various mechanisms of these assays, suggesting that combined assay methods should be adopted in the screening and evaluation of bioactive compounds from natural materials.

Our experimental results also showed that the substitution position of the caffeoyl group also influenced free radical scavenging activity. By analyzing the structure of similar compounds and experimental results, we found that the free radical scavenging activity of 3-caffeoyl substitution was optimal, followed by 4-caffeoyl substitution and 6-caffeoyl substitution. This conclusion could be supported by the order of activity as follows: **1** > **2** > **4**, **7** > **8**.

### Protective effects against free radical-induced oxidative damage

Repeated ultraviolet B (UVB) exposure or constant treated with Fe^2+^/H_2_O_2_ can produce reactive oxygen species (ROS), which lead to various adverse effects on the body tissues^40^,^41^. The free radicals could attack on the fatty acid component of membrane lipids, and then resulting in mitochondrial damage and lipid peroxidation. These facts have been previously demonstrated in mitochondria^42^. Therefore, UVB-induced oxidative damage model and Fe^2+^/H_2_O_2_-induced oxidative damage model are classical mitochondria models which are used to measure the protective effect against free radical-induced oxidative damage.

When mitochondria are damaged, swelling will occur; thus, the value of A_520_ will be reduced^43^. Malondialdehyde (MDA) and lipid hydroperoxide (LOOH) are two relatively unstable products of lipid peroxidation, which is a process where ROS degrade polyunsaturated fatty acids^41^. These toxic products could cause toxic stress in mitochondria, accelerate further oxidative damage^44^. Antioxidant enzymes, such as catalase (CAT), glutathione reductase (GR), and superoxide dismutase (SOD) can confer protection against oxidative stress and tissue damage. These enzymes are critical for defense against the harmful effects of ROS and free radicals in biological systems^45^. In this study, compounds **1**–**9** were investigated for their protective effects against free radical-induced oxidative damage.

**Figure 4:**
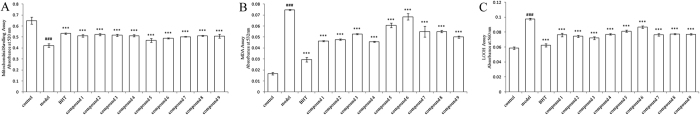
Protective activity of nine compounds against UVB-induced formation of mitochondrial swelling degree (**A**), MDA (**B**), and LOOH (**C**) in rat liver mitochondria. Data are presented as the mean ± SD (*n* = 3). Symbols represent statistical significance. ***p < 0.001, compared with the model group; ^###^*p* < 0.001, compared with the control group. BHT is butylated hydroxytoluene.

**Figure 5:**
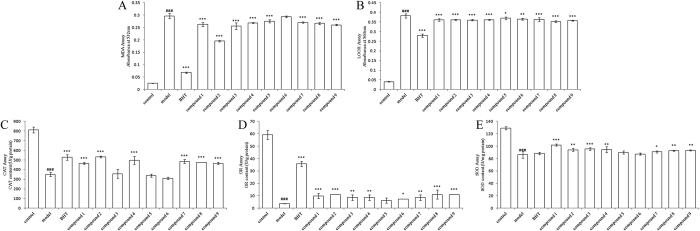
Protective activity of nine compounds against Fe^2+^/H_2_O_2_-induced formation of MDA (**A**), LOOH (**B**), CAT (**C**), GR (**D**) and SOD (**E**) in rat liver mitochondria. Data are presented as the mean ± SD (*n* = 3). Symbols represent statistical significance. ***p < 0.001, compared with the model group; **p < 0.01, compared with the model group; *p < 0.05, compared with the model group; ^###^*p* < 0.001, compared with the control group. BHT is butylated hydroxytoluene.

In mitochondrial damage model caused by UVB (Fig. 4), the model group showed a significant decrease in mitochondrial swelling assay, and the change was significantly reversed during treatment with test compounds (*p* < 0.001). The A_532_ value and A_560_ value of the MDA (0.075 ± 0.001) and LOOH (0.097 ± 0.001) were significantly increased in the model group compared with the control group (*p* < 0.001). However, the test compound groups reduced the MDA and LOOH level and showed significant effects (*p* < 0.001). In mitochondrial damage model caused by Fe^2+^/H_2_O_2_, compared with the control group, the model group showed a significant decrease in CAT, GR and SOD levels, together with a significant increase in the level of MDA and LOOH (Fig. 5). Overall, these changes were significantly reversed during treatment with test compounds. However, the level of improvement of some group was not significant (*p* > 0.05).

Comparing the above experimental results, we found that protective effect of compounds containing two pair of two adjacent phenolic groups was optimal, followed by compounds containing one pair of two adjacent phenolic groups (such as compounds **5** and **6**). Furthermore, compounds with two sugars were best. These results were consistent with the experimental results of free radical scavenging assays. Therefore, we could speculate that the protective effects of the nine phenylethanoid glycosides against free radical-induced oxidative damage were attributed to their radical scavenging activity, which was caused by the number of two adjacent phenolic groups.

### Potential of *M. officinalis* var. *biloba* fruits as a promising functional alternative food resource

Oral malodor is a major social and psychological problem that affects the majority of the general population^20^. Oral malodor is divided into two kinds, namely, pathological and physiological. Pathological oral malodor is mainly caused by oral diseases, such as caries. Physiological oral malodor is the result of lipid peroxidation from gastrointestinal food debris. Studies^2^,^4^,^20^ have proven that the antioxidant and antimicrobial activities of magnolol and honokiol are responsible for oral malodor combat. In the present study, phenylethanoid glycosides were demonstrated to have free radical scavenging activities and inhibitory effects of lipid peroxidation. Thus, phenylethanoid glycosides could assist in improving the condition of physiological oral malodor. *M. officinalis* var. *biloba* fruits should be used as an alternative food resource added in gum and mints. More studies are necessary to further understand the action mechanism of phenylethanoid glycosides. These studies should also add further value to this by-product of *M. officinalis* var. *biloba* fruits.

## Methods

### Plant material

*M. officinalis* var. *biloba* fruits were collected from the mountain area in Enshi (N: 30°17′51.56″, E: 109°28′27.33″; A: 1,545 m), Hubei Province, China, and identified by Prof. Youwei Wang (Wuhan University, Wuhan, China). A voucher specimen (No. 14610) was deposited at the Traditional Chinese Medicine Specimen Museum, School of Pharmaceutical Science, Wuhan University, China.

### Chemical and reagents

2,2′-Azinobis (3-ethylbenzthiazoline-6-sulphonic acid) (ABTS) was procured from Fluka (Menlo Park, CA, USA). 2,2-Diphenyl-1-picrylhydrazyl (DPPH), high-performance liquid chromatography (HPLC)-grade methanol, and acetonitrile were purchased from Sigma–Aldrich Co. (St. Louis, USA). D-Allose (D1218014), L-rhamnose (B1504021), L-cysteine methyl ester hydrochlorides (G14118068), and 2-thiobarbituric acid (C1510117) were purchased from Aladdin Industrial Corporation (Shanghai, China). Diagnostic kits for CAT, GR, and SOD were purchased from Nanjing Jiancheng Technology Co. Ltd. (Nanjing, China). Ascorbic acid (Vc) (F20060609), ammonium iron (II) sulfate hexahydrate (20120215), butylated hydroxytoluene (BHT) (20121211), D-glucose (20140724), dodecyl sodium sulfate (20130625), hydrogen peroxide 30% (20150911), iron (II) sulfate heptahydrate (20100506), methanol-*d*_*4*_ (20140519), xylenol orange (20100826), and other analytical chemicals and reagents were purchased from Sinopharm Chemical Regent Co. Ltd. (Shanghai, China).

### Instrumentation

All NMR spectra (^1^H NMR^13^,C NMR, H-H COSY, HMQC, and HMBC spectra) were obtained on a Bruker DPX-400 spectrometer using standard Bruker pulse programs (Bruker BioSpin GmbH, Rheinstetten, Germany) with solvent peak as references. HR-ESI-MS data were obtained on a Bruker Daltonic micrOTOF-Q II MS spectrometer (Bruker BioSpin GmbH, Rheinstetten, Germany). UV absorption spectra were recorded on a UV-2600 ultraviolet and visible spectrophotometer (Shimadzu, Tokyo, Japan) from 200 nm to 900 nm. Infrared (IR) spectra were recorded on a Nicolet Nexus 470 spectrometer (Nicolet, Madison, American) using the compounds in the KBr pellet form. Preparative HPLC was carried out on a LabAllance Model 201 HPLC system (LabAllance, Tianjin, China) and a Kromasil C18 column preparative column (10 μm, 250 × 30 mm, Kromasil, Stockholm, Sweden).

### Preparation of plant extracts

The dried and powdered *M. officinalis* var. *biloba* fruits (5.1 kg, size less than 0.25 mm) were extracted three times using 10 volumes of 70% alcohol (50 L) for 3 h at reflux. The filtrate was combined and concentrated under vacuum to yield a dried alcohol extract (804.9 g). The alcohol extract was suspended in distilled water (10 L) and then successively partitioned three times with chloroform (20 L), ethyl acetate (10 L), and *n*-butanol alcohol (10 L), which yielded the chloroform fraction (398.4 g), ethyl acetate fraction (180.8 g), and *n*-butanol fraction (63.4 g), respectively. All fractions were stored at −20 °C until further use.

### Isolation and purification procedures of phenylethanoid glycosides

The *n*-butanol fraction (20 g) prepared from the previously described steps was further isolated on a preparative HPLC system. Each chromatographic run was carried out at a flow rate of 20 mL/min with a binary mobile phase consisting of methanol (A) and 0.1% formic acid (B) using a step gradient profile. The gradient started with 10% A, was varied to 35% A at 10 min, 46% A at 40 min, 48% A at 43 min, 100% A at 50 min, and 100% A isocratic for 10 min, and decreased to 10% A in 0.1 min. After re-equilibration at 10% A for 12 min, the next sample was injected. The temperature of the column oven was 25 °C, and 100 μL (300 mg/mL) was injected into the system every time. The peaks adsorbed at 315 nm were recorded. Compounds **1** (797.7 mg), **2** (226.4 mg), **3** (195.9 mg), and **4** (298.3 mg) were obtained at the retention times of 22.4, 32.6, 35.3, and 41.7 min, respectively. The subfraction that was collected at a retention time from 27 min to 29 min was further applied to the preparative HPLC system, which was eluted isocratically with 18% acetonitrile in water (containing 0.1% formic acid) at a flow rate of 20 mL/min. Compounds **5** (56.0 mg) and **6** (34.1 mg) were then purified. Simultaneously, the subfraction obtained at a retention time of 19.3 min was subjected to the preparative HPLC system, which was eluted isocratically with 26% methanol in water at a flow rate of 20 mL/min. Compounds **7** (37.7 mg) and **8** (52.7 mg) were then obtained. The subfraction obtained at a retention time of 38.4 min was further isolated over the preparative HPLC system, which was eluted isocratically with 43% methanol in water at a flow rate of 20 mL/min to yield compound **9** (35.9 mg).

### Acid hydrolysis and sugar analysis in glycosides

Sugar analysis of compounds **2**, **3**, **5**, **6** and **8** was carried out according to the method described by previous studies^46^,^47^. In brief, compounds **2**, **3**, **5**, **6** and **8** (each 4.0 mg) were hydrolyzed by heating in 4 M CF_3_COOH (4 mL) at 95 °C for 4 h. After cooling, the solution was extracted with CH_2_Cl_2_ (3 × 2 mL). The water layer was concentrated by reducing pressure and dried by vacuum. Anhydrous pyridine (0.5 mL) and L-cysteine methyl ester hydrochloride (2 mg) were added and reacted at 60 °C for 1 h. After cooling to room temperature, o-tolyl isothiocyanate (10 μL) was added to the mixture and further heated at 60 °C for 1 h. The reaction mixture was directly analyzed by reversed-phase HPLC using a Diamonsil C18 analytical column (5 μm, 250 × 4.6 mm), which was eluted isocratically with 25% acetonitrile in water (containing 0.1% formic acid) at a flow rate of 1 mL/min. The temperature of the column oven was 25 °C, and 20 μL was injected into the system every time. The UV spectra were collected at 250 nm. Peaks of the hydrolysate of compounds **2**, **3**, **5**, **6** and **8** were identified by comparing the retention times of authentic samples of D-allose (t_R_ = 19.330), D-glucose (t_R_ = 18.566), and L-rhamnose (t_R_ = 33.411) after simultaneous treatment under the same conditions.

### DPPH and ABTS radical scavenging assays

DPPH and ABTS, two relatively stable free radical compounds widely used to test free radical scavenging activity, were determined using a previously described method^48^,^49^. Radical scavenging activity was calculated using the equation: radical scavenging activity (%) = [(A_0_ − A)/A_0_] × 100 (where A_0_ is the absorbance of the control, and A is the absorbance of the test sample). V_C_ and BHT with the same concentrations as the samples were used as positive controls.

### Superoxide anion radical (O_2_^−^) scavenging assay

The superoxide anion radical is the most common free radical generated *in vivo*. The capacity of each compound to scavenge superoxide radicals was examined by a pyrogallol auto-oxidation system^50^. Pyrogallic acid can auto-oxidize under alkaline conditions to produce O_2_^−^ directly, and the rate constant of this auto-oxidation reaction is dependent on the O_2_^−^ concentration. If the test compound can scavenge O_2_^−^, the auto-oxidation reaction can slow down significantly. The auto-oxidation rate constant (K_b_) of pyrogallic acid was calculated from the curve of A_325 _nm versus time. V_C_ and BHT were used for comparative purposes. Simultaneously, the K_b_ value (10^−4 ^A/s) of the blank control was recorded.

### Test animals

Sprague–Dawley (SD) rats (200 ± 20 g) were purchased from the Laboratory Animal Center of Wuhan University, Wuhan, China. All the rats were housed under regulated conditions of 12 h light/12 h dark cycles, 25 ± 2 °C, and 30%–60% relative humidity. They were fed with a standard pellet diet (rat feed, purchased from the Laboratory Animal Center of Wuhan University, Wuhan, China) and unlimited water. The animals were allowed to acclimatize to the room conditions for 2 days. All experimental procedures were approved by the Institutional Animal Ethical Committee of the Committee for the Purpose of Control and Supervision of Experiments on Animals, Wuhan University in Wuhan, China. The experimental methods were performed in accordance with “Regulations for the Administration of Affairs Concerning Experimental Animals” and “Guiding Opinions of Treating Experimental Animals with Good Ethics”, which have been formally released and implemented by Chinese Government.

### Preparation of mitochondria

Mitochondria were isolated from SD rats according to the method described by previous studies^45^. All procedures were carried out at 4 °C.

### Mitochondrial damage model caused by UVB

The experiment consisted of the control group, model group, BHT group, and test sample group. Each group comprised the following set up: for the control group, 0.5 mL of mitochondrial protein + 0.1 mL of phosphate buffer (0.05 M, pH 7.4); for the model group, 0.5 mL of mitochondrial protein + 0.1 mL of phosphate buffer (0.05 M, pH 7.4) + UVB; for the BHT group, 0.5 mL of mitochondrial protein + 0.1 mL of BHT (12.5 μM, in phosphate buffer) + UVB; and for the test compound group, 0.5 mL of mitochondrial protein + 0.1 mL of test sample (12.5 μM, in phosphate buffer) + UVB. The model, BHT, and test compound groups were irradiated for 4 h with a 20 W UVB lamp (TL/12RS, Philips) from a 15 cm distance. The control group was kept at 37 °C for 4 h without irradiation.

### Mitochondrial damage model caused by Fe^2+^/H_2_O_2_

The experiment consisted of the control group, model group, BHT group, and test sample group. Each group comprised the following set up: for the control group, 0.5 mL of mitochondrial protein + 0.5 mL of phosphate buffer (0.05 M, pH 7.4); for the model group, 0.5 mL of mitochondrial protein + 0.5 mL of phosphate buffer (0.05 M, pH 7.4) + 30 μL FeSO_4_(10 mM) + 20 μL H_2_O_2_ (65 mM); for the BHT group, 0.5 mL of mitochondrial protein + 0.5 mL of BHT (12.5 μM, in phosphate buffer) + 30 μL FeSO_4_(10 mM) + 20 μL H_2_O_2_ (65 mM); and for the test compound group, 0.5 mL of mitochondrial protein + 0.1 mL of test sample (12.5 μM, in phosphate buffer) + 30 μL FeSO_4_(10 mM) + 20 μL H_2_O_2_ (65 mM). All experiment groups were kept at 37 °C for 1 h.

### Measurement of biochemical indicators

The values of A_520_ were used to evaluate the mitochondrial swelling degree^43^. MDA assay was evaluated using the thiobarbituric acid method^45^. LOOH assay was measured using a modified version of a previously reported method^45^. CAT, GR and SOD activities in mitochondrial protein were measured by commercial kits purchased from Nanjing Jiancheng Technology Co. Ltd. (Nanjing, China).

### Statistical analysis

All experiments were carried out in triplicate, and the results were reported as the mean ± standard deviation (*n* = 3). The data were analyzed using one-way ANOVA. Statistically significant effects were analyzed, and the means were also compared using least-significant difference (LSD) test. Statistical significance was determined at *p* < 0.05.

## Additional Information

**How to cite this article**: Ge, L. *et al*. Nine phenylethanoid glycosides from *Magnolia officinalis* var. *biloba* fruits and their protective effects against free radical-induced oxidative damage. *Sci. Rep.* **7**, 45342; doi: 10.1038/srep45342 (2017).

**Publisher's note:** Springer Nature remains neutral with regard to jurisdictional claims in published maps and institutional affiliations.

## Supplementary Material

Supplementary Information

## Figures and Tables

**Figure 1:**
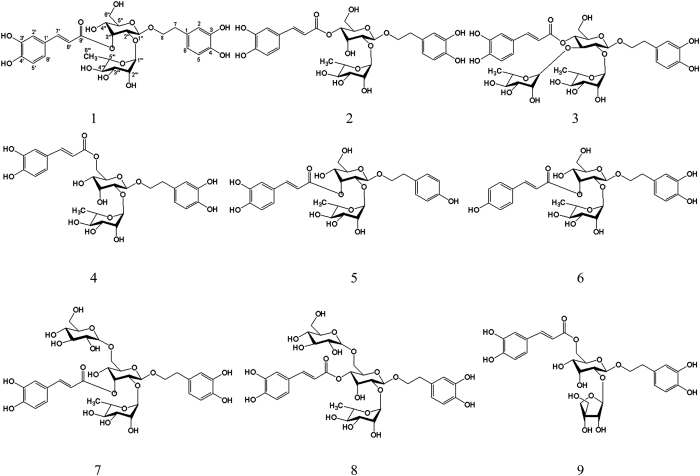
Chemical structure of all identified phenylethanoid glycosides in *M. officinalis* var. *biloba* fruits.

**Table 1:** ^1^H-NMR (CD_3_OD, 400 MHz) data of compounds 2, 8, and 3 (*δ*_H_, dppm).

Position	^1^H-NMR		
	2	8	3
	Aglycone		
1	—	—	—
2	6.73 (1H,d,2.0)	6.74 (1H,d,2.0)	6.71 (1H,d,2.0)
3	—	—	—
4	—	—	—
5	6.71 (1H,d,8.0)	6.71 (1H,d,8.0)	6.69 (1H,d,8.0)
6	6.58 (1H,dd,2.0,8.0)	6.60 (1H,dd,2.0,8.0)	6.58 (1H,dd,2.0,8.0)
7	2.80 (2 H,t,7.4)	2.80 (2 H,t,7.4),	2.84 (2 H,t,8.0),
8	3.94 (1H,m);3.77 (1H,m)	3.65 (1H,m);3.76 (1H,m)	3.69 (1H,m);3.91 (1H,m)
	Caffeoyl		
1′	—	—	—
2′	7.08 (1H,d,2.0)	7.09 (1H,d,2.0)	7.07 (1H,d,2.0)
3′	—	—	—
4′	—	—	—
5′	6.80 (1H,d,8.0)	6.81 (1H,d,8.0)	6.80 (1H,d,8.0)
6′	6.97 (1H,dd,2.0,8.0)	6.97 (1H,dd,2.0,8.0)	6.97 (1H,dd,2.0,8.0)
7′	7.63 (1H,d,15.9)	7.63 (1H,d,15.9)	7.60 (1H,d,15.9)
8′	6.33 (1H,d,15.9)	6.33 (1H,d,15.9)	6.30 (1H,d,15.9)
9′	—	—	—
	Allose		Glucose
1″	4.85 (1H,d,8.0)	4.85 (1H,d,8.0)	4.51 (1H,d,7.7)
2″	3.47 (1H,m)	3.46 (1H,m)	3.97 (1H,m)
3″	4.51 (1H,s)	4.51 (1H,s)	3.57 (1H,m)
4″	4.84 (1H,m)	4.92 (1H,m)	4.96 (1H,m)
5″	3.60 (1H,m)	3.22 (1H,m)	3.51 (1H,m)
6″	3.62 (1H,m); 3.79 (1H,m)	3.67 (1H,m); 4.01 (1H,m)	3.52 (1H,m); 3.63 (1H,m)
	2″-Rhamnose		
1″′	4.90 (1H,s)	4.89 (1H,s)	4.96 (1H,s)
2″′	3.68 (1H,m)	4.02 (1H,m)	3.46 (1H,m)
3″′	3.61 (1H,m)	4.24 (1H,m)	3.84 (1H,m)
4″′	3.36 (1H,m)	3.93 (1H,m)	3.32 (1H,m)
5″′	4.04 (1H,m)	3.67 (1H,m)	4.05 (1H,m)
6″′	1.29 (3H,d,6.0)	1.30 (3H,d,6.0)	1.14 (3H,d,6.0)
		6″-Glucose	3″-Rhamnose
1″″		4.30 (1H,d,7.7)	5.01 (1H,s)
2″″		3.60 (1H,m)	3.46 (1H,m)
3″″		3.25 (1H,m)	3.84 (1H,m)
4″″		3.32 (1H,m)	3.32 (1H,m)
5″″		3.35 (1H,m)	4.05 (1H,m)
6″″		3.64 (1H,m); 3.86 (1H,m)	1.29 (3H,d,6.0)

**Table 2:** ^13^C-NMR (CD_3_OD, 100 MHz) data of compounds **2**, **8**, and **3** (*δ*_C_, dppm).

Position	^13^C-NMR		
	2	8	3
Aglycone			
1	131.68	131.67	130.70
2	117.25	116.63	115.87
3	146.82	146.80	146.32
4	144.61	144.59	144.17
5	116.59	117.31	116.64
6	121.41	121.51	120.78
7	36.73	36.72	36.04
8	72.30	72.45	71.86
Caffeoyl			
1′	127.70	127.68	127.17
2′	115.30	115.37	114.74
3′	146.01	145.97	145.61
4′	149.75	149.77	149.26
5′	116.45	116.53	116.04
6′	123.20	123.33	122.70
7′	147.72	147.96	147.38
8′	114.71	114.66	114.47
9′	168.14	168.23	167.91
Allose			Glucose
1″	100.47	100.50	102.46
2″	73.19	73.97	82.68
3″	66.45	66.42	80.43
4″	70.57	70.64	69.83
5″	74.53	75.03	75.50
6″	62.37	69.62	61.88
		2″-Rhamnose	
1″′	97.91	97.91	103.07
2″′	72.37	72.19	72.01
3″′	72.22	72.25	71.71
4″′	73.99	72.36	73.33
5″′	69.99	70.01	70.71
6″′	18.04	18.09	17.91
		6″-Glucose	3″-Rhamnose
1″″		104.92	102.32
2″″		74.41	71.80
3″″		77.78	71.49
4″″		71.48	73.18
5″″		77.90	70.43
6″″		62.67	17.69

**Table 3:** ^1^H-NMR (CD_3_OD, 400 MHz) and ^13^C-NMR (CD_3_OD, 100 MHz) data of compounds 5 and 6.

Position	5		6	
	δ_H_	δ_C_	δ_H_	δ_C_
Aglycone				
1	—	130.80	—	131.60
2	7.10 (1H,d,2.0)	131.02	6.72 (1H,d,2.0)	117.17
3	6.71 (1H,d,8.0)	116.18	—	146.91
4	—	156.74	—	144.62
5	6.71 (1H,d,8.0)	116.18	6.69 (1H,d,8.0)	116.37
6	7.10 (1H,d,2.0)	131.02	6.59 (1H,dd,2.0,8.0)	121.37
7	2.85 (2 H,t,8.0)	36.57	2.79 (2 H,t,8.0)	36.75
8	3.67 (2 H,m)	72.19	3.67 (2 H,m)	72.15
	Caffeoyl		Coumaroyl	
1′	—	127.82	—	127.24
2′	7.08 (1H, d,2.0)	115.31	7.51 (1H,d,8.0)	131.30
3′	—	146.84	6.84 (1H,d,8.0)	116.88
4′	—	149.66	—	161.30
5′	6.81 (1H,d,8.0)	116.58	6.84 (1H,d,8.0)	116.88
6′	6.99 (1H,dd,2.0,8.0)	123.13	7.51 (1H,d,8.0)	131.30
7′	7.61 (1H,d,15.9)	147.33	7.68 (1H,d,15.9)	146.04
8′	6.38 (1H,d,15.9)	115.18	6.45 (1H,d,15.9)	115.23
9′	—	168.93	—	168.89
Allose				
1″	4.76 (1H,d,8.0)	100.86	4.76 (1H,d,8.0)	100.80
2″	3.65 (1H,m)	73.86	3.65 (1H,m)	73.89
3″	5.75 (1H,t,3.0)	71.22	5.75 (1H,t,3.0)	69.95
4″	3.76 (1H,m)	67.17	3.76 (1H,m)	67.15
5″	3.82 (1H,m)	76.06	3.82 (1H,m)	76.03
6″	3.69 (1H,m); 3.92 (1H,m)	62.74	3.70 (1H,m); 3.92 (1H,m)	62.71
Rhamnose				
1″′	4.94 (1H,s)	98.49	4.95 (1H,s)	98.44
2″′	3.6–3.8 (1H,m)	72.31	3.6–3.8 (1H,m)	72.25
3″′	3.6–3.8 (1H,m)	72.05	3.6–3.8 (1H,m)	72.02
4″′	3.42 (1H,m)	73.80	3.42 (1H,m)	73.79
5″′	4.02 (1H,m)	69.99	4.02 (1H,m)	71.19
6″′	1.25 (3H,d,6.0)	18.07	1.26 (3H,d,6.0)	18.02

**Table 4:** Radical scavenging activities of phenylethanoid glycosides from *M. officinalis* var. *biloba* fruits.

Compound	IC_50_ (μM)		K_b_ ( × 10^−4^ A/s)
	DPPH	ABTS	Superoxide anion
**1**	11.79 ± 0.57	3.52 ± 0.11	10.37 ± 0.33
**2**	12.99 ± 0.48	3.53 ± 0.28	10.68 ± 0.49
**3**	21.38 ± 0.52	3.28 ± 0.35	11.57 ± 0.17
**4**	16.23 ± 0.16	3.75 ± 0.11	10.50 ± 0.18
**5**	32.18 ± 0.97	4.16 ± 0.25	11.22 ± 0.23
**6**	35.17 ± 0.22	5.09 ± 0.07	9.84 ± 0.38
**7**	22.94 ± 0.26	4.61 ± 0.10	9.41 ± 0.77
**8**	24.62 ± 0.15	4.78 ± 0.08	9.07 ± 0.76
**9**	20.99 ± 0.50	6.23 ± 0.06	8.69 ± 0.70
Vc	40.94 ± 0.78	11.51 ± 1.05	0.09 ± 0.02
BHT	89.94 ± 4.57	7.29 ± 0.33	10.40 ± 0.55
control	—	—	15.35 ± 0.19

Values were the means ± SD, n = 3; Vc is ascorbic acid; BHT is butylated hydroxytoluene.
