# Supplementary material for: Nine phenylethanoid glycosides from Magnolia officinalis var. biloba fruits and their protective effects against free radical-induced oxidative damage
Source: Sci Rep. 2017 Mar 28;7:45342. doi: 10.1038/srep45342 (PMC5368604; doi:10.1038/srep45342)
Supplement: Supplementary Information [file srep45342-s1.pdf]

Nine phenylethanoid glycosides from *Magnolia officinalis* var. *biloba* fruits and their protective effects against free radical-induced oxidative damage

Lanlan Ge,[1] Wenhui Zhang,[1] Gao Zhou,[1] Bingxin Ma,[1] Qigui Mo,[1] Yuxin Chen,[1]

Youwei Wang[*1,2]

[1]Institute of TCM and Natural Products, School of Pharmaceutical Sciences, Wuhan University, Wuhan 430071, P. R. China. [2]MOE Key Laboratory of Combinatorial Biosynthesis and Drug Discovery, Wuhan University, Wuhan 430072, P. R. China. Correspondence and requests for materials should be addressed to Y.W. (email: wyw@whu.edu.cn).

[*] Corresponding author. Tel.: +86 27 68759323; Fax: +86 27 68759010.

E-mail address: wyw@whu.edu.cn

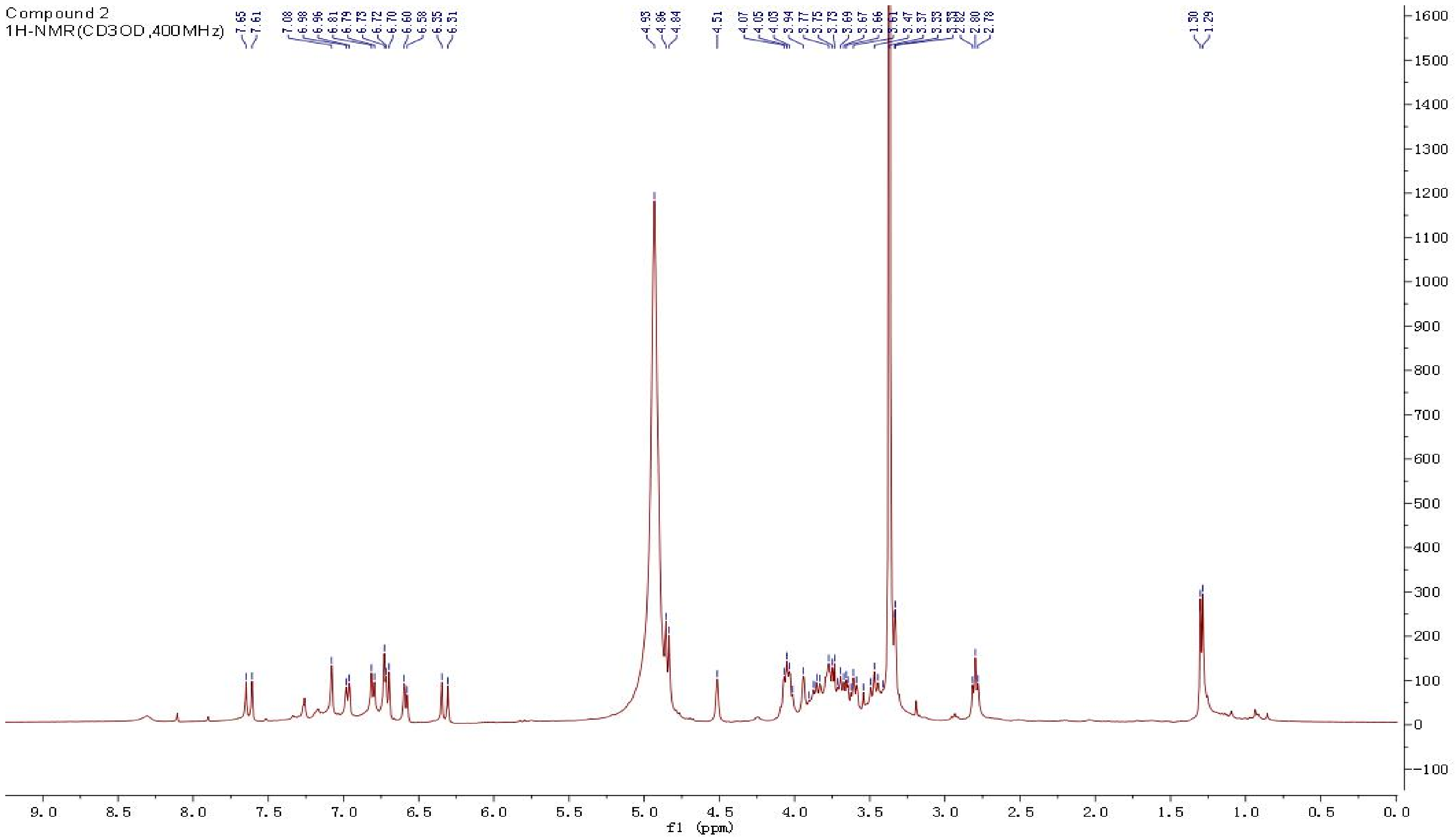

^{1}H-NMR spectrum of compound 2

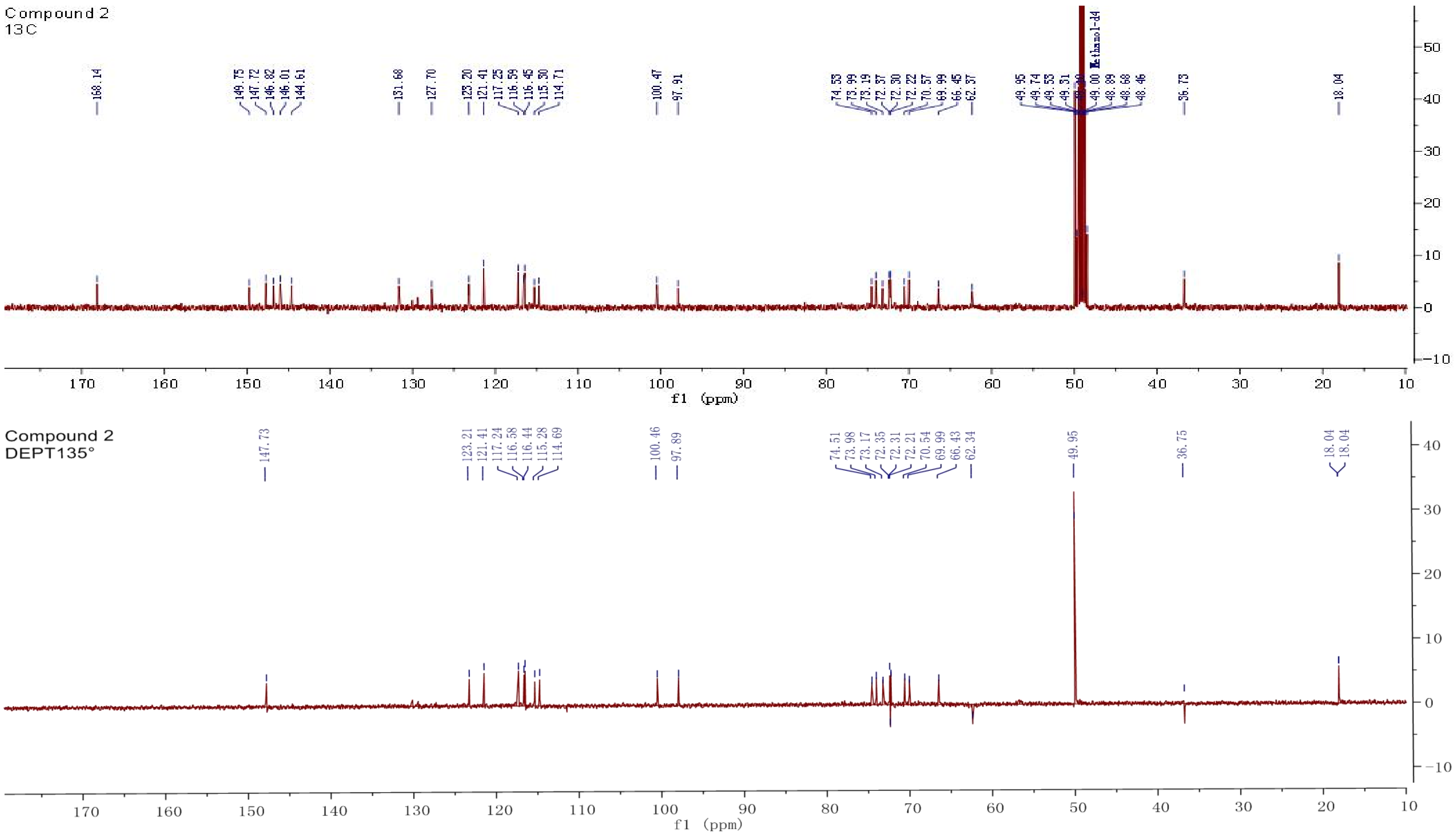

^{13}C-NMR spectrum of compound 2

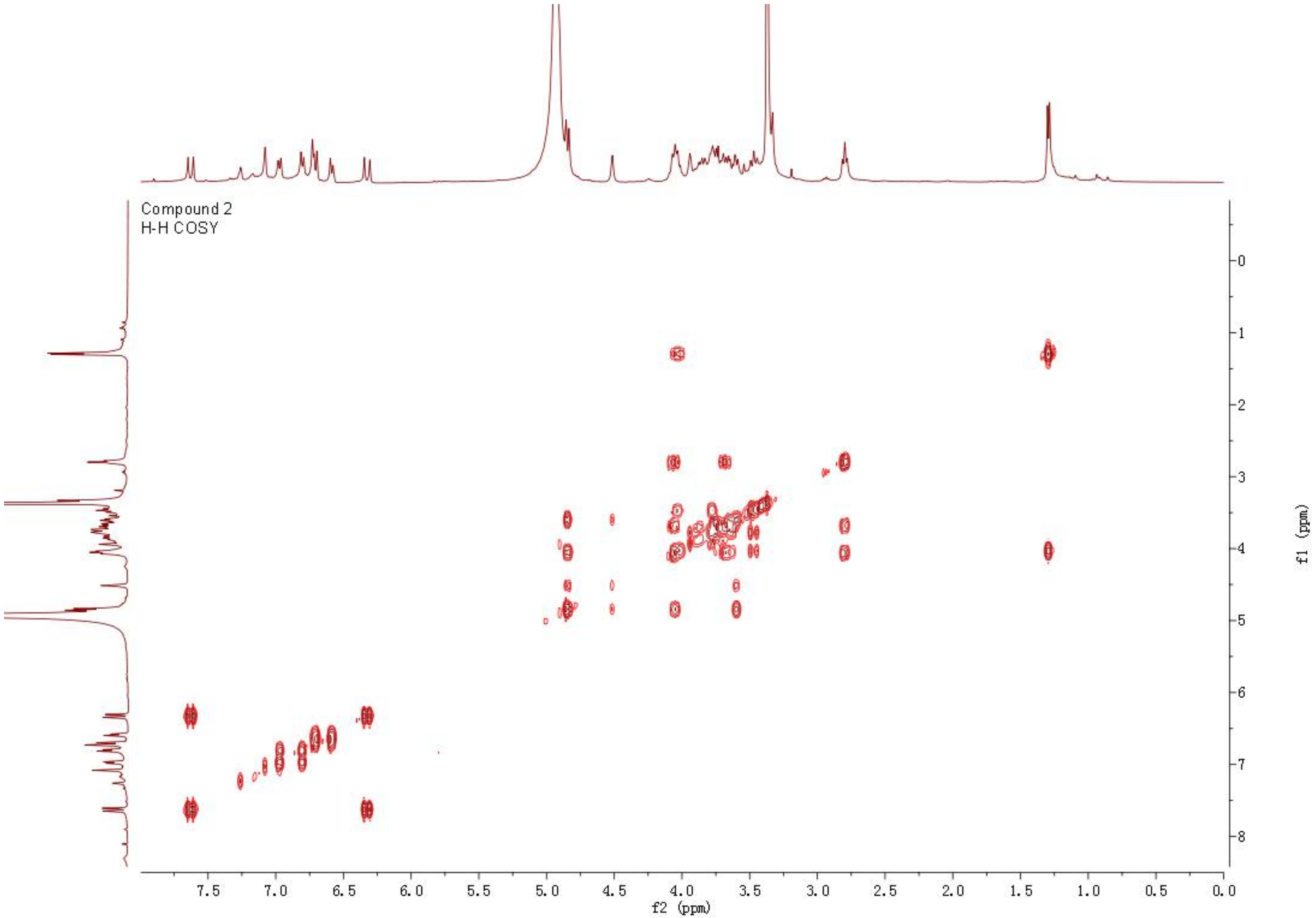

H-H COSY spectrum of compound 2

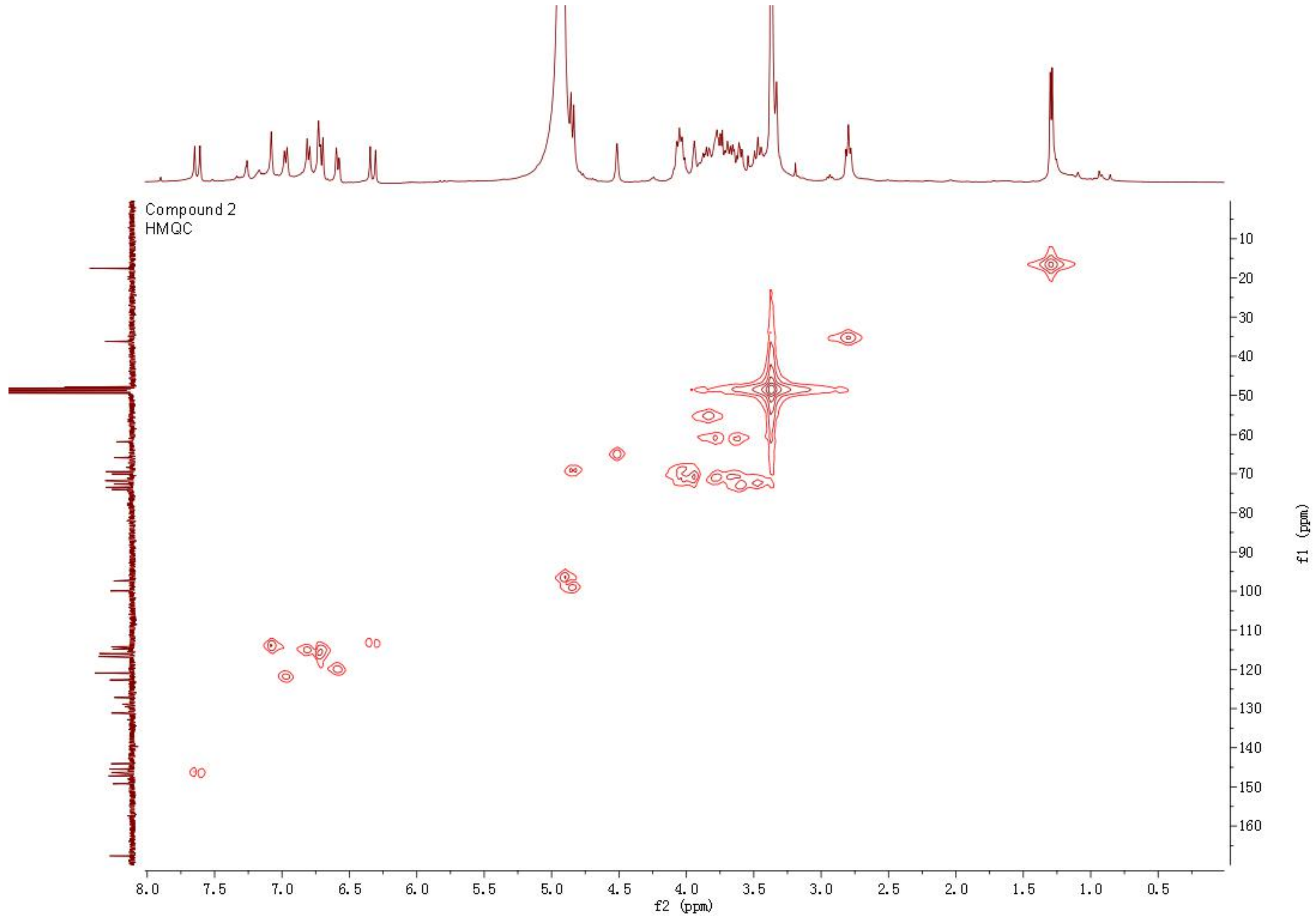

HMQC spectrum of compound 2

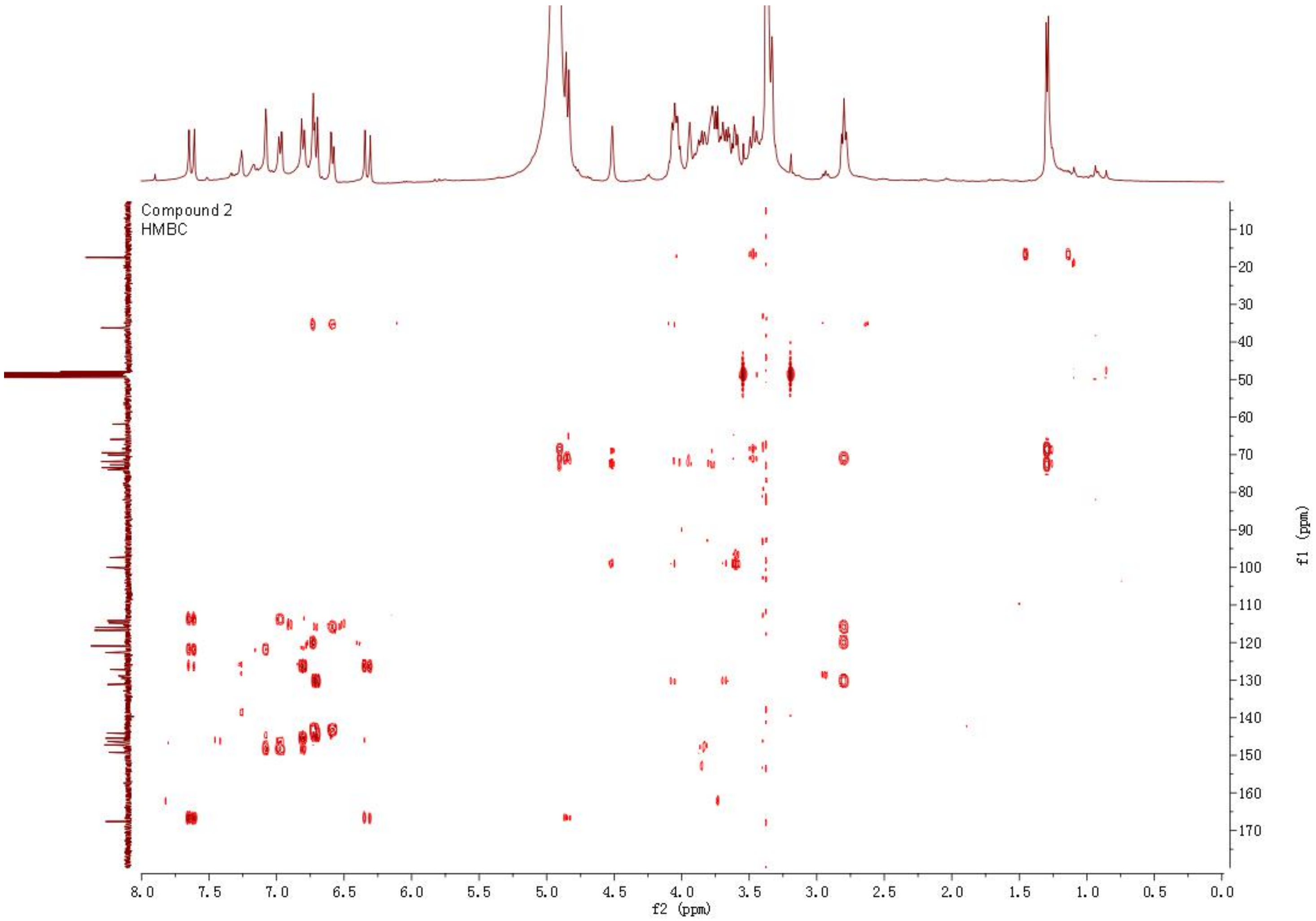

HMBC spectrum of compound 2

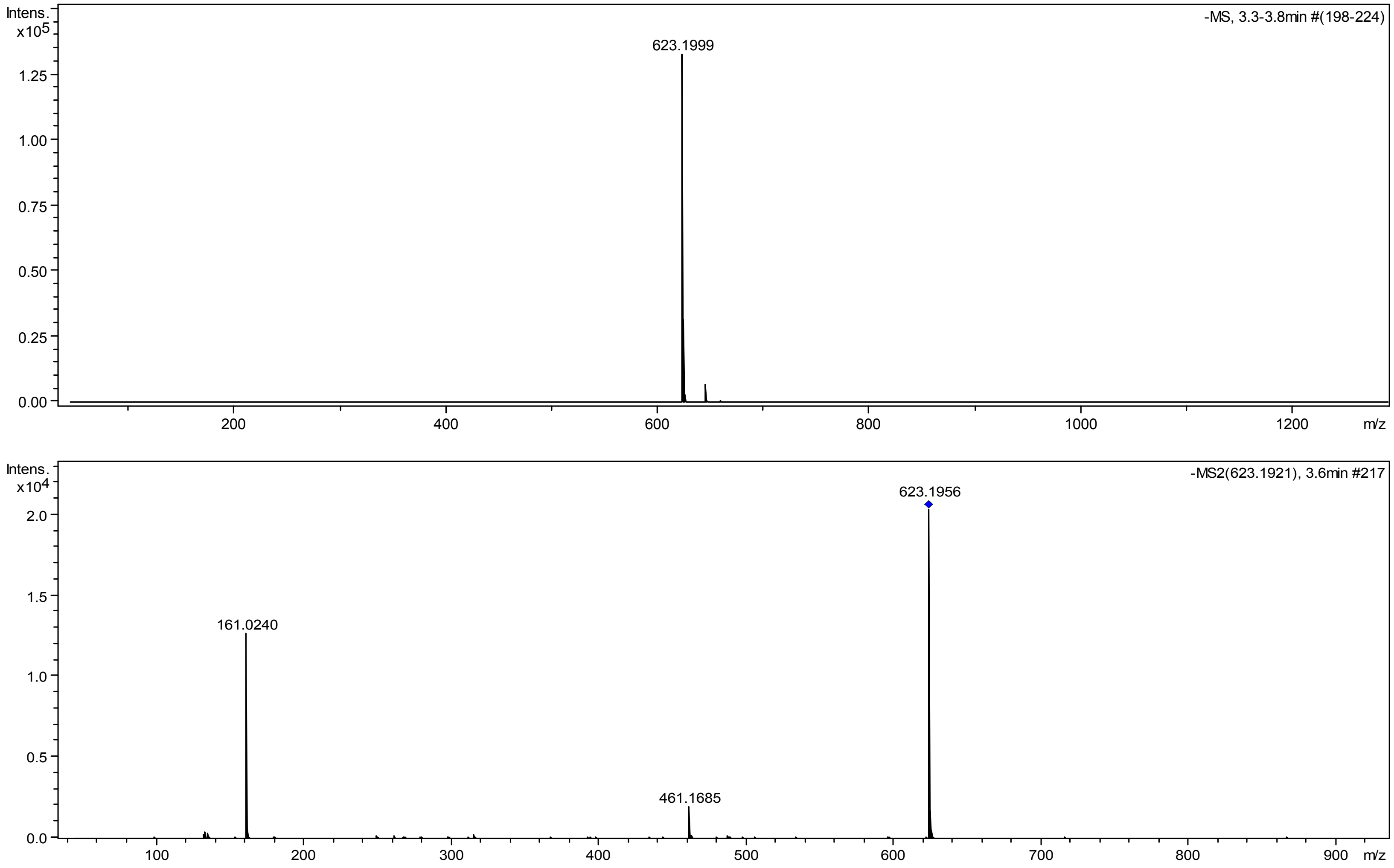

HR- ESI-MS f compound 2

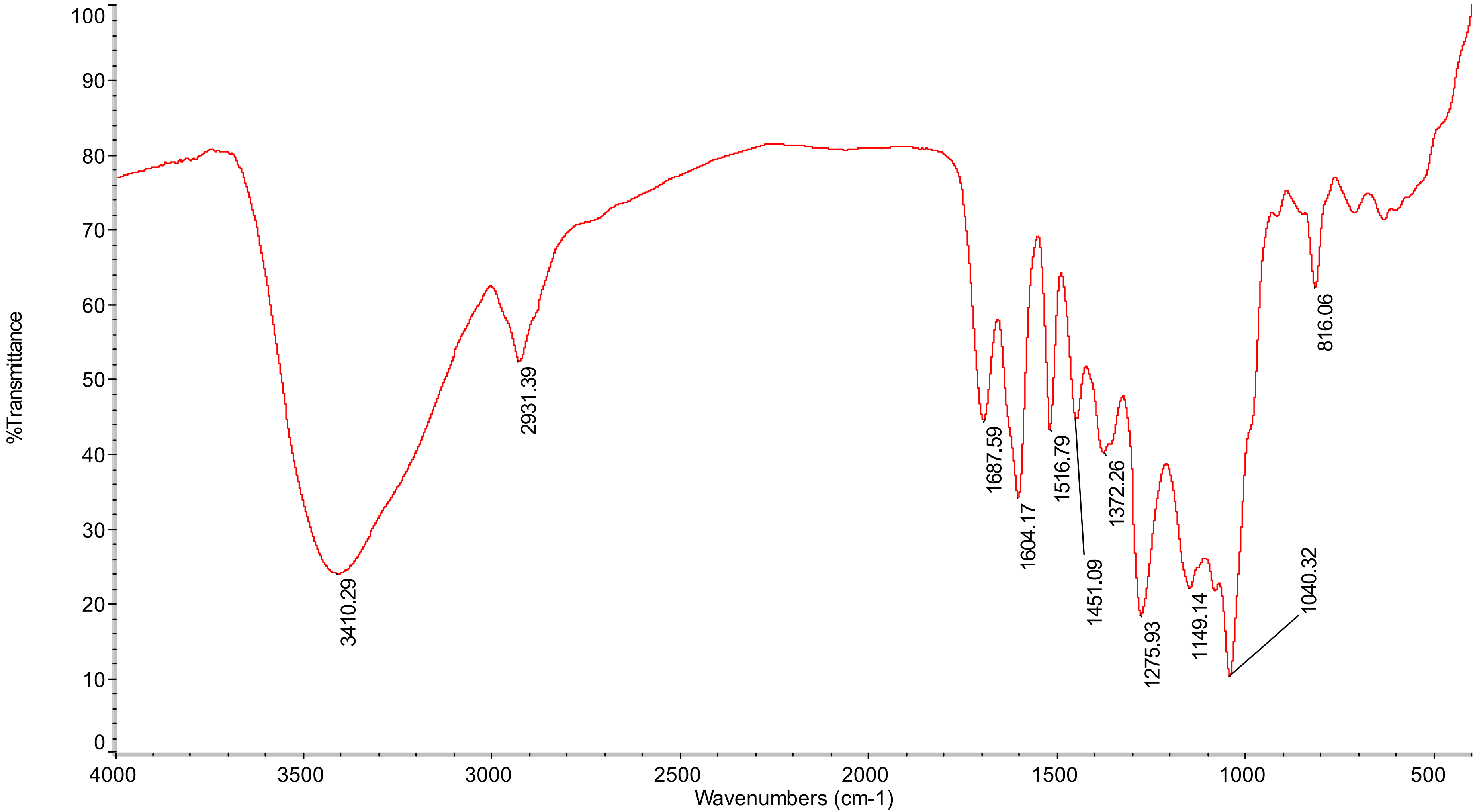

IR spectrum of compound 2

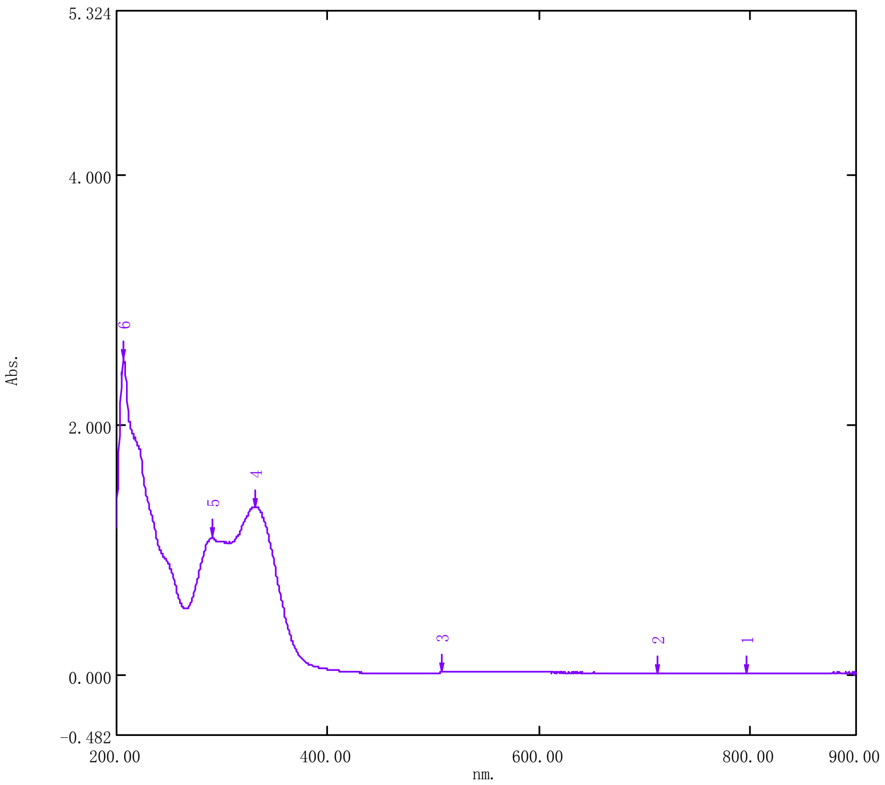

UV scan of compound 2

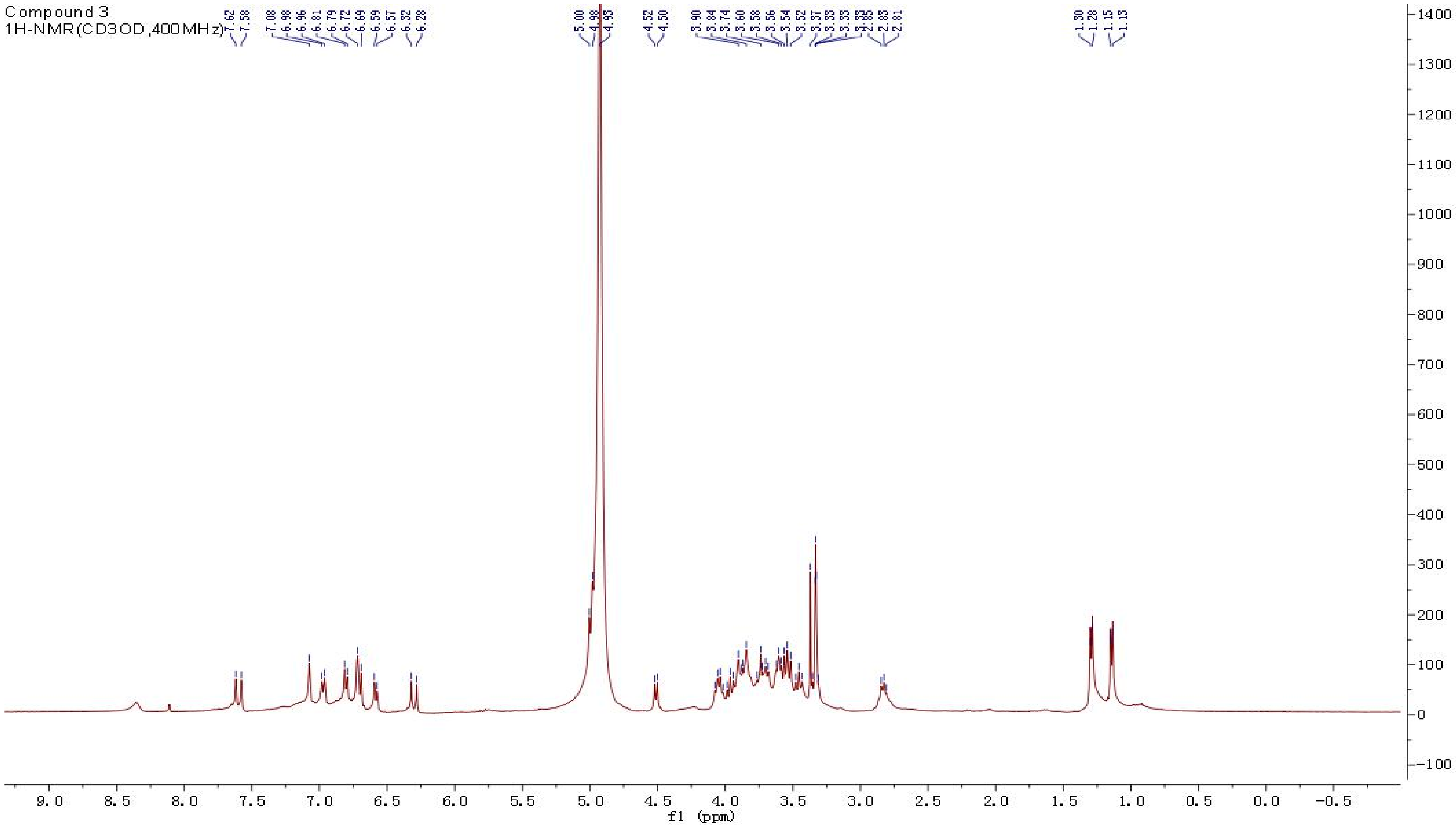

^{1}H-NMR spectrum of compound 3

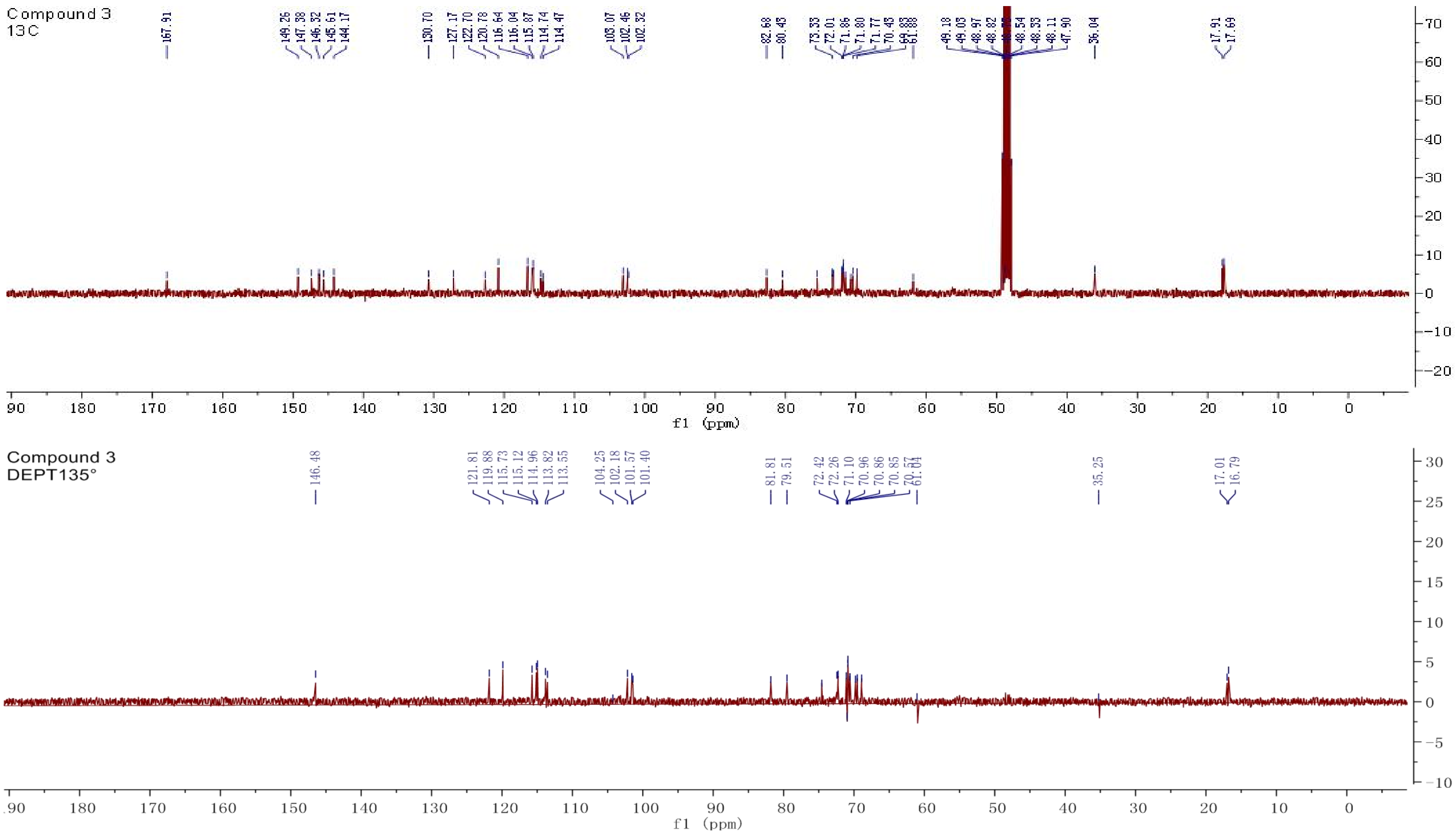

^{13}C-NMR spectrum of compound 3

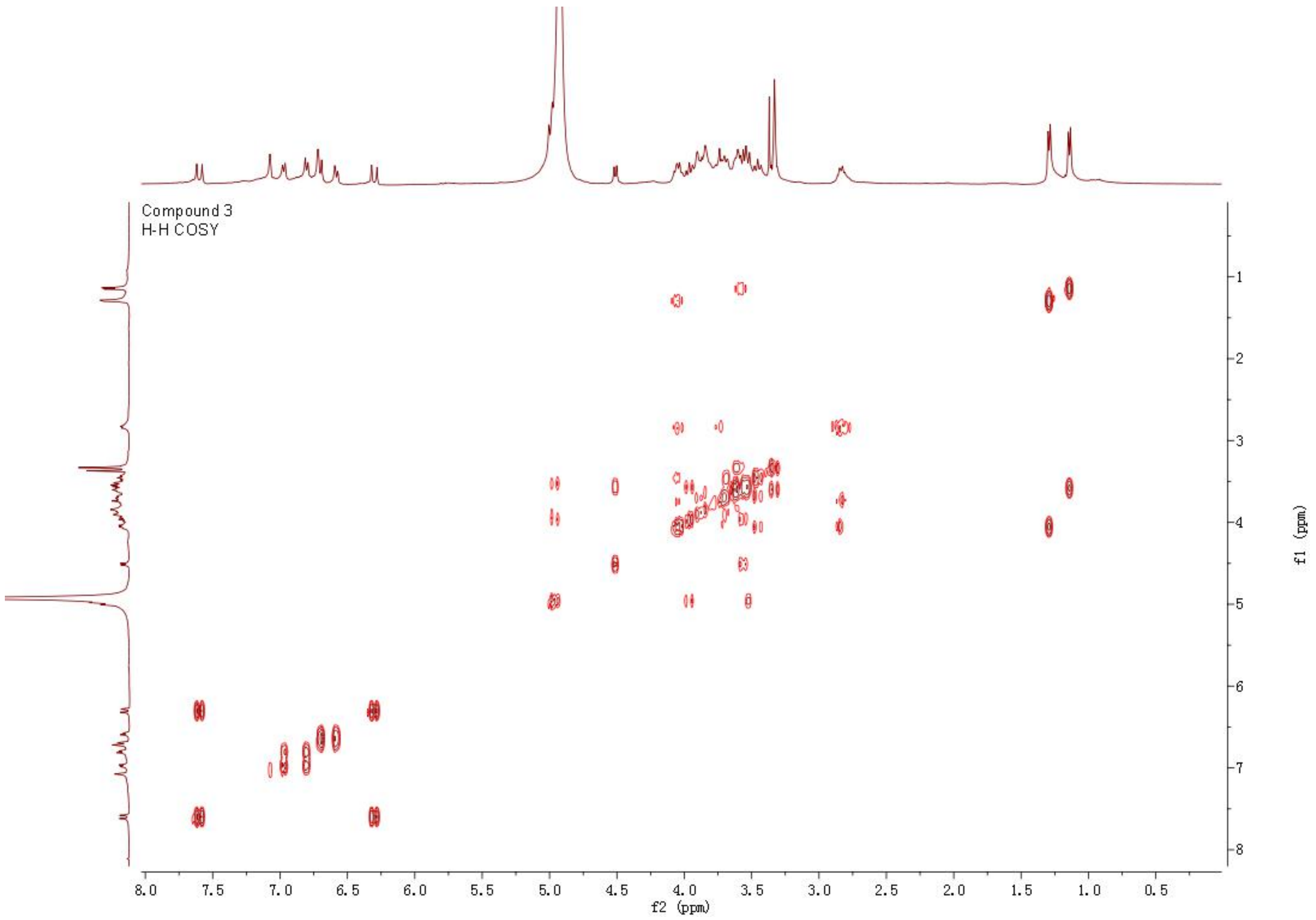

H-H COSY spectrum of compound 3

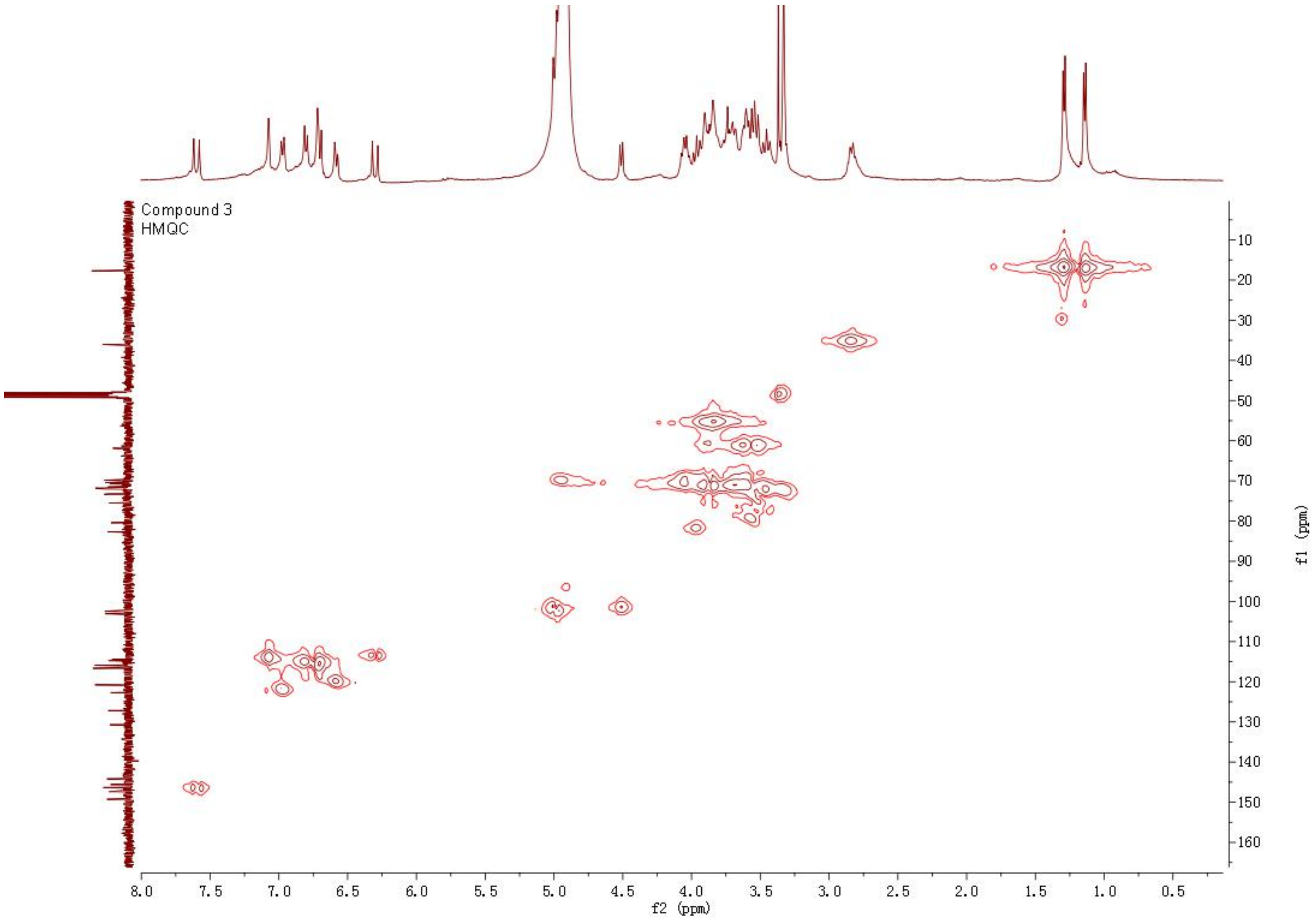

HMQC spectrum of compound 3

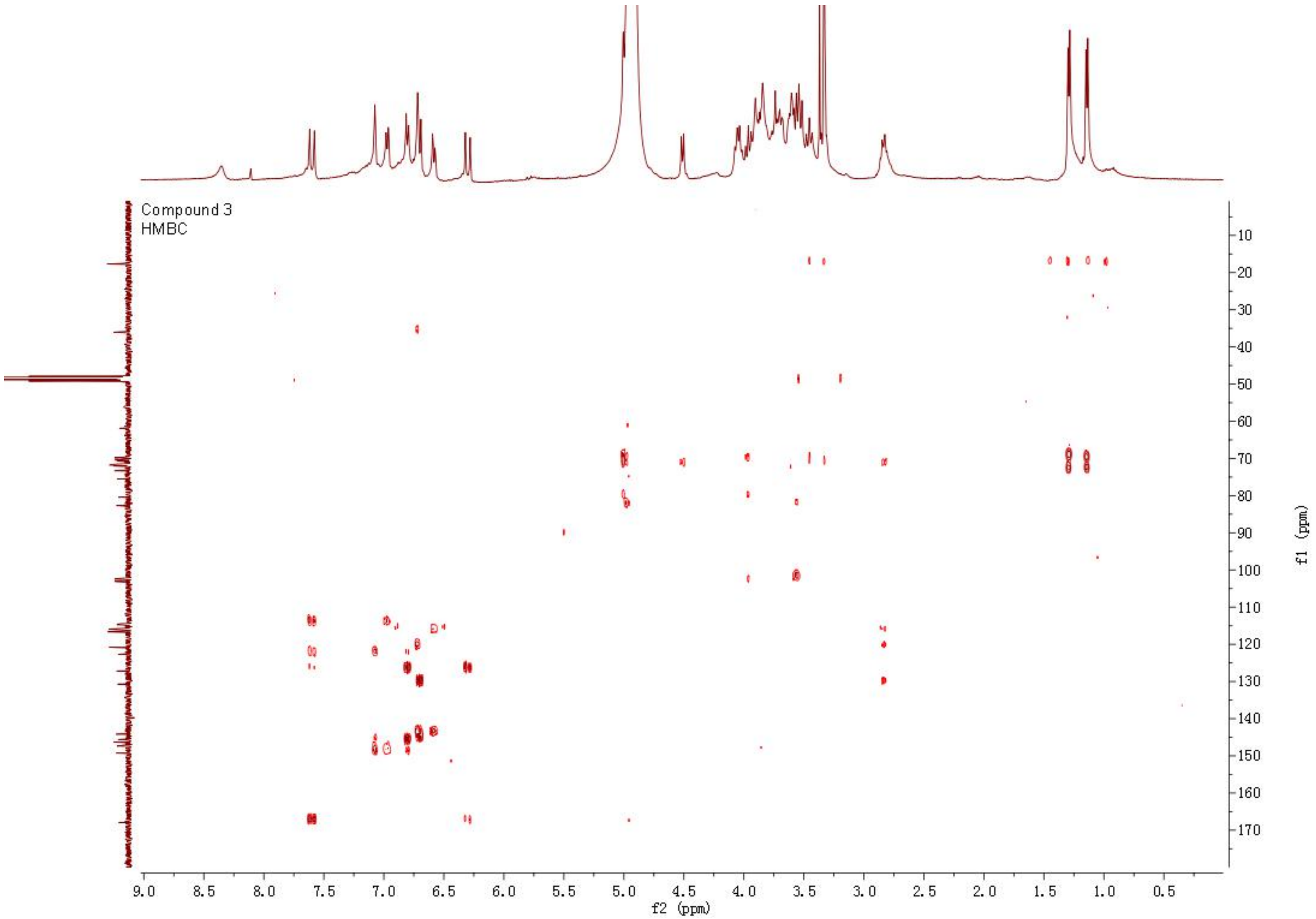

HMBC spectrum of compound 3

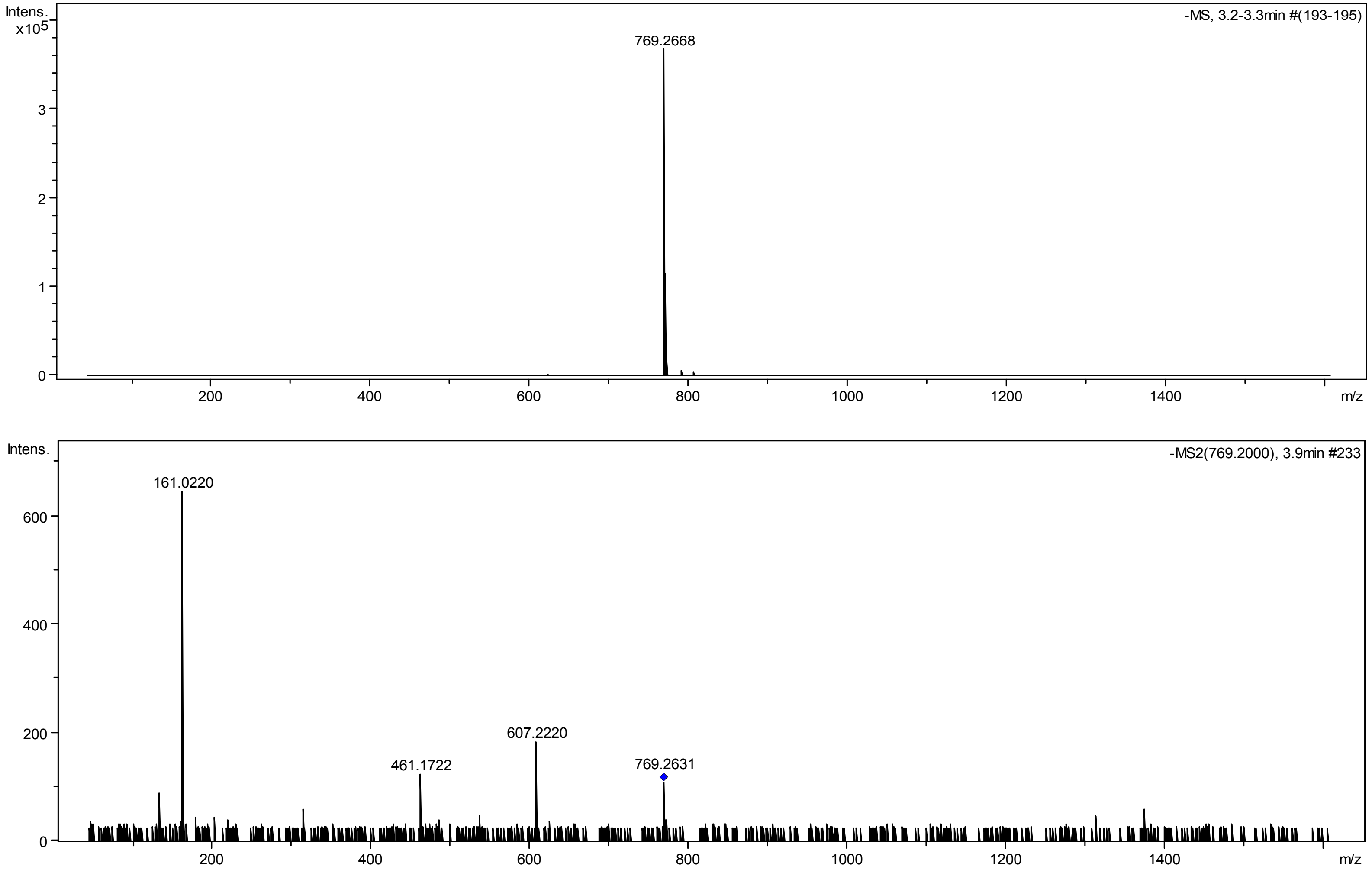

HR- ESI-MS f compound 3

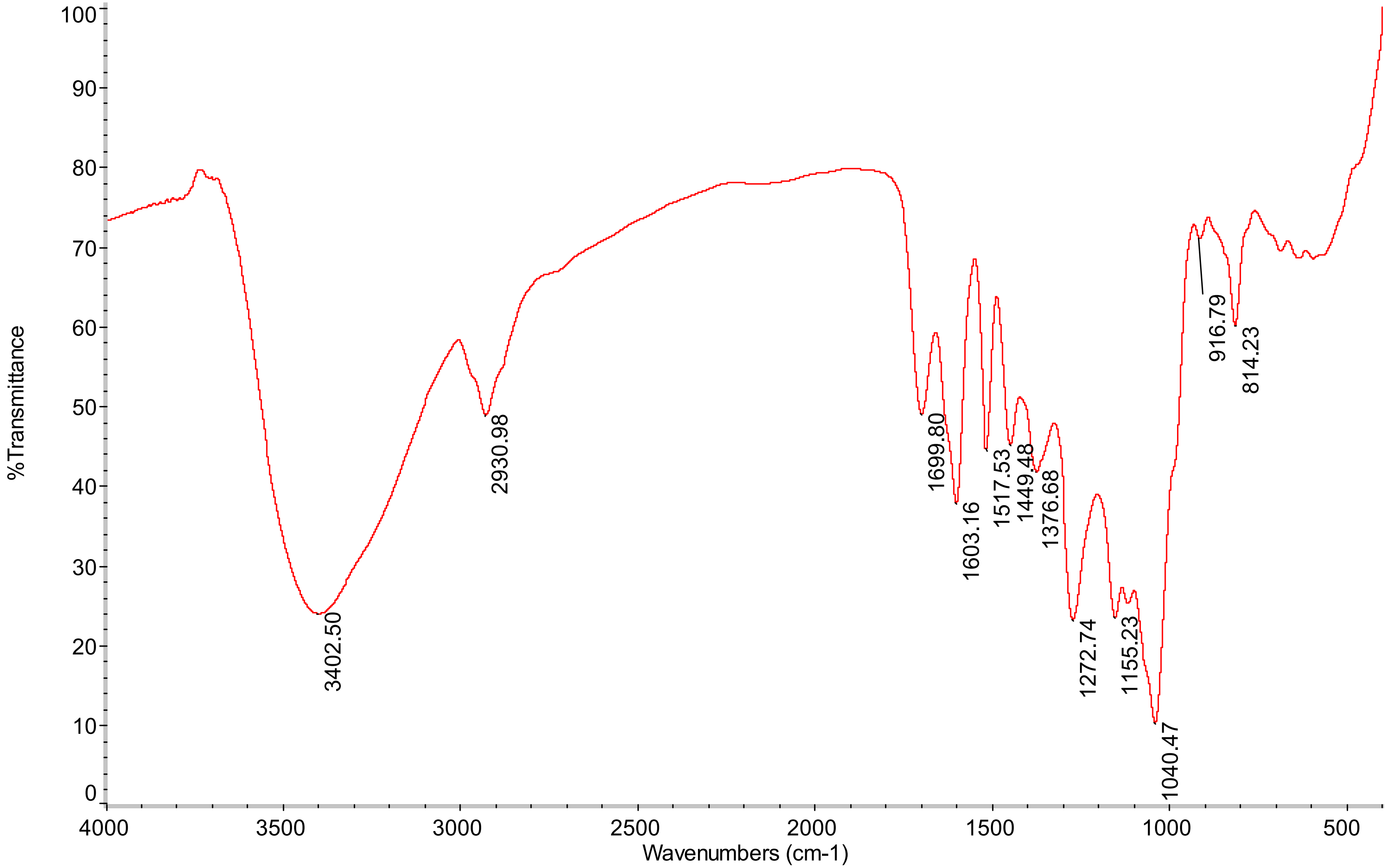

IR spectrum of compound 3

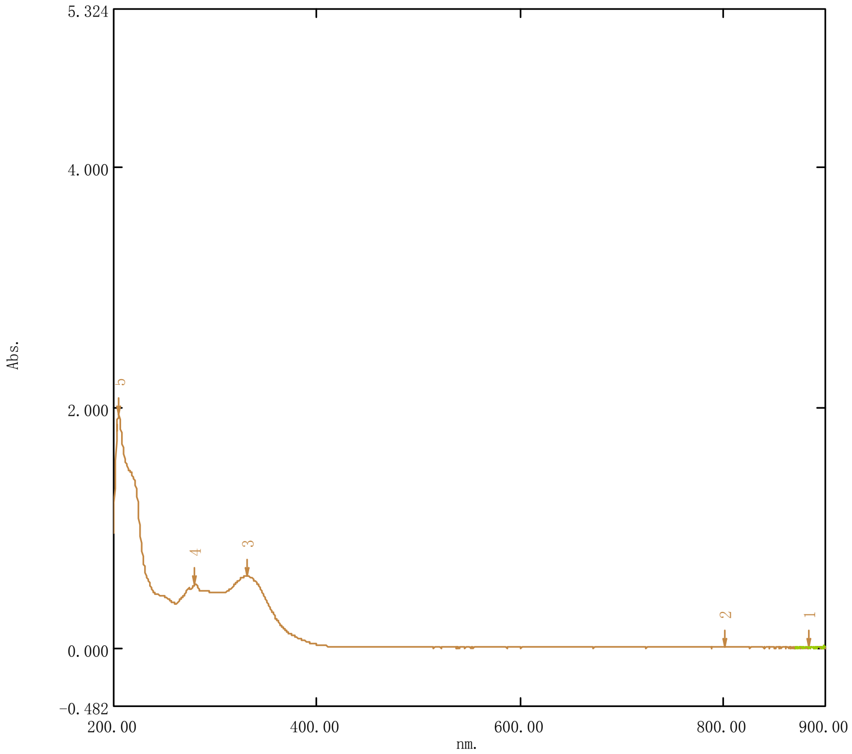

UV scan of compound 3

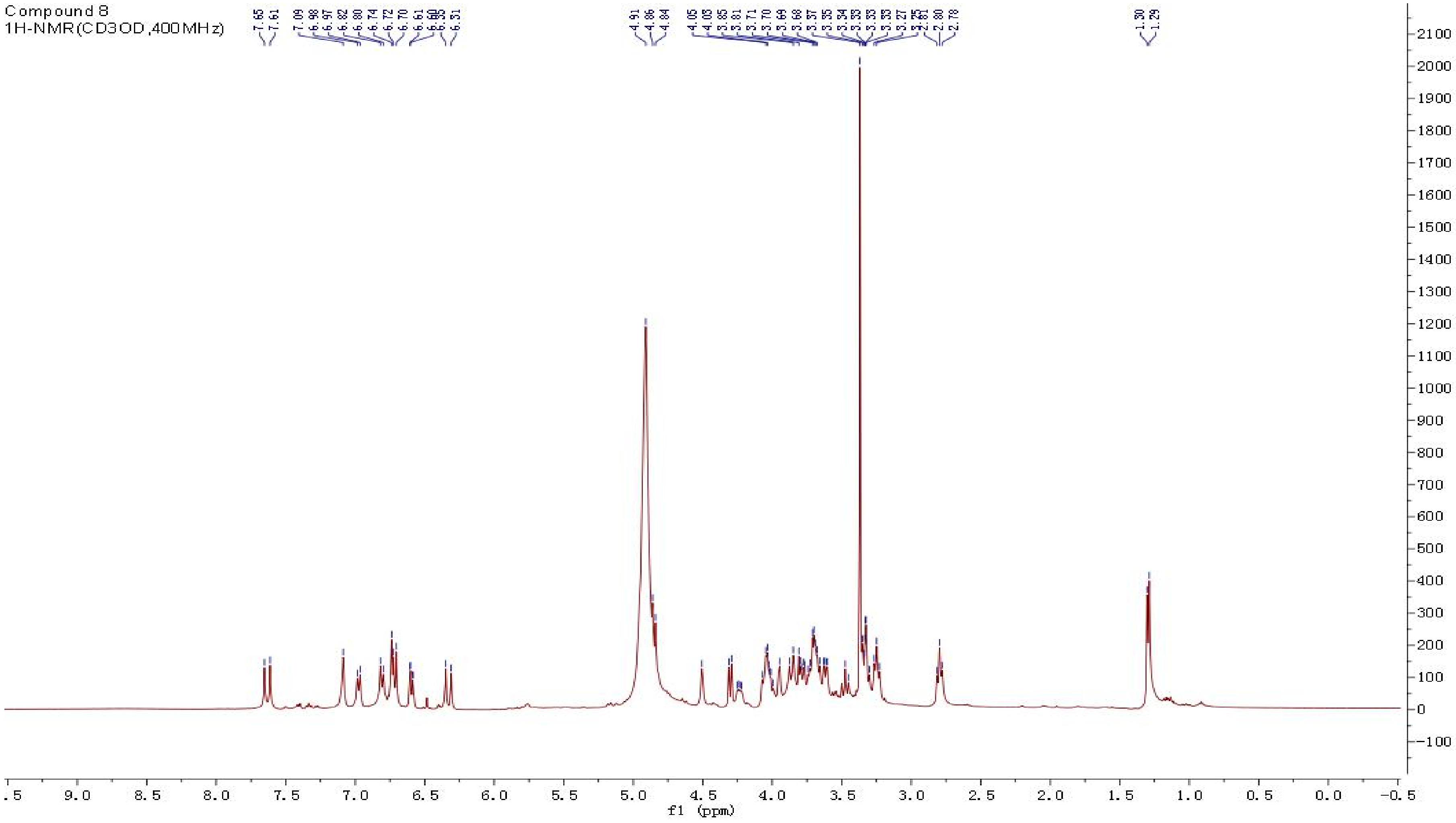

^{1}H-NMR spectrum of compound 8

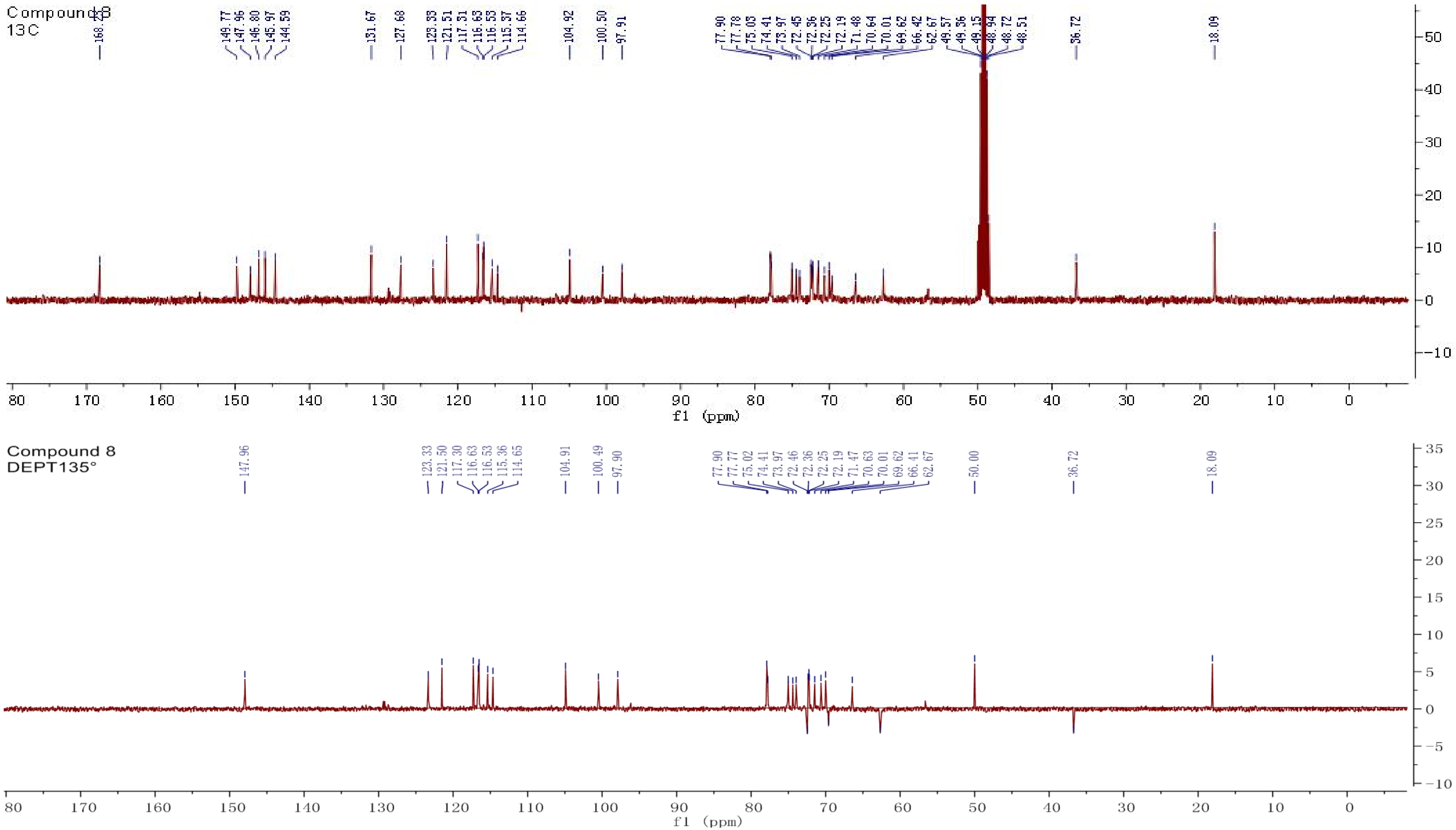

^{13}C-NMR spectrum of compound 8

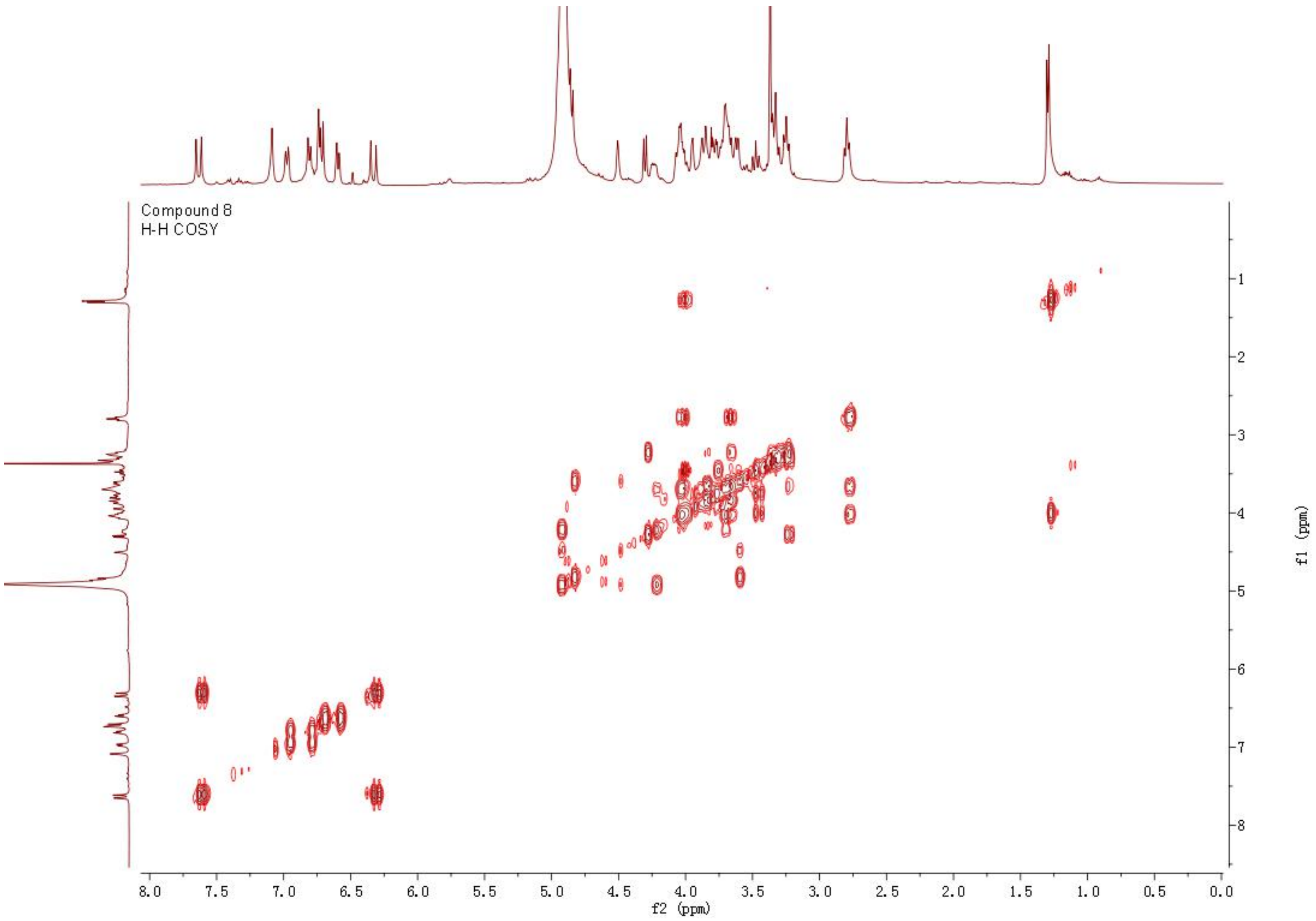

H-H COSY spectrum of compound 8

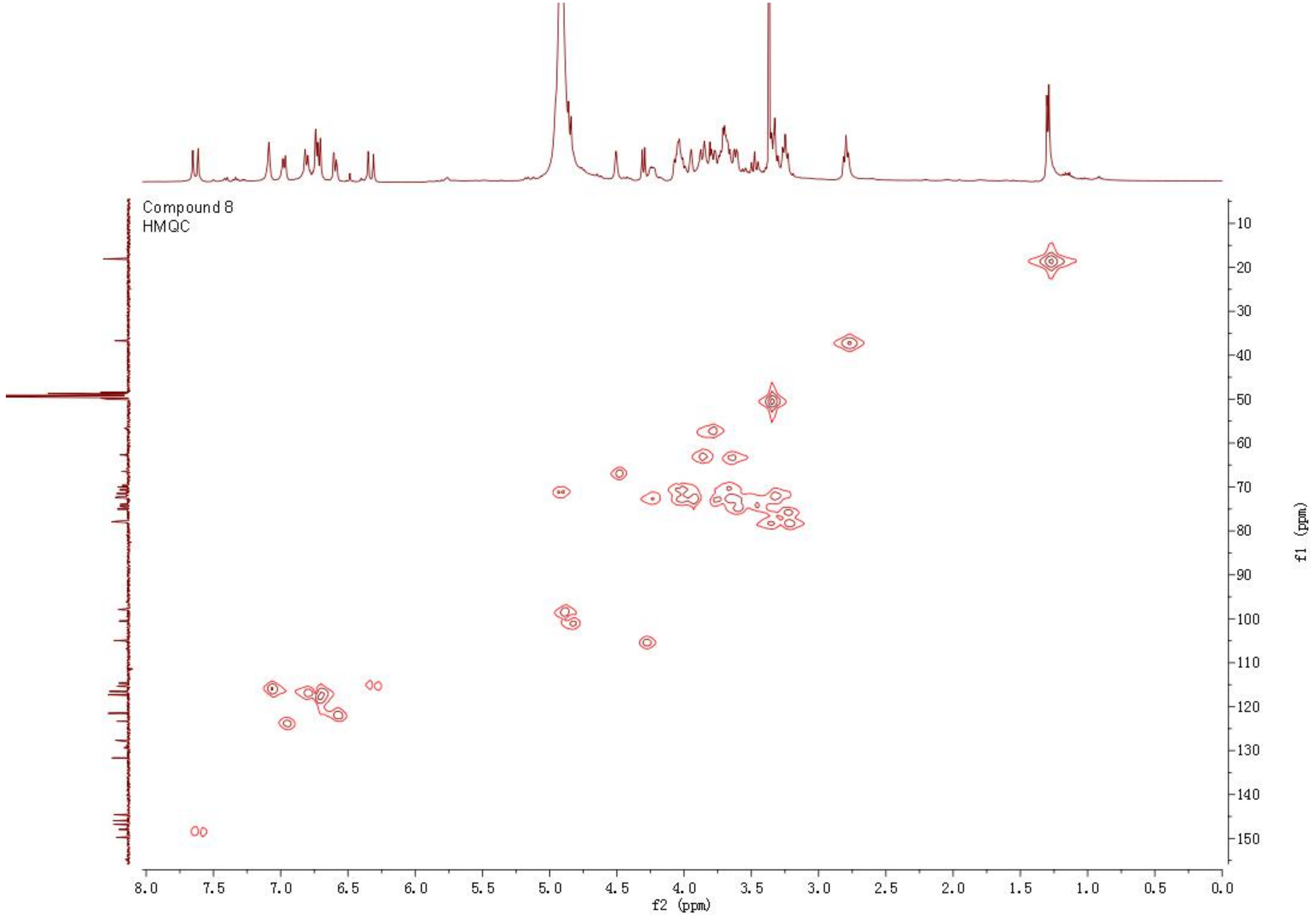

HMQC spectrum of compound 8

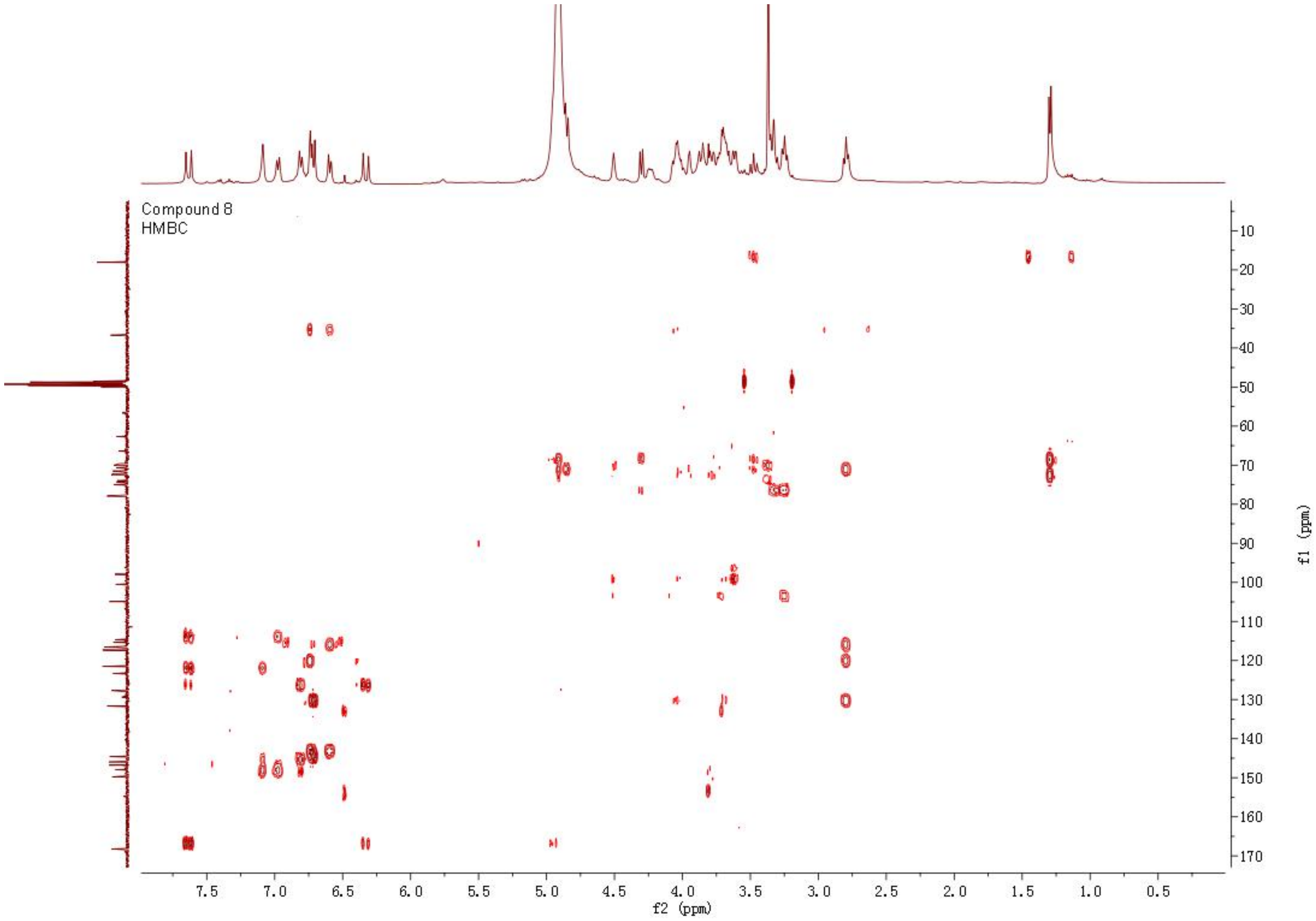

HMBC spectrum of compound 8

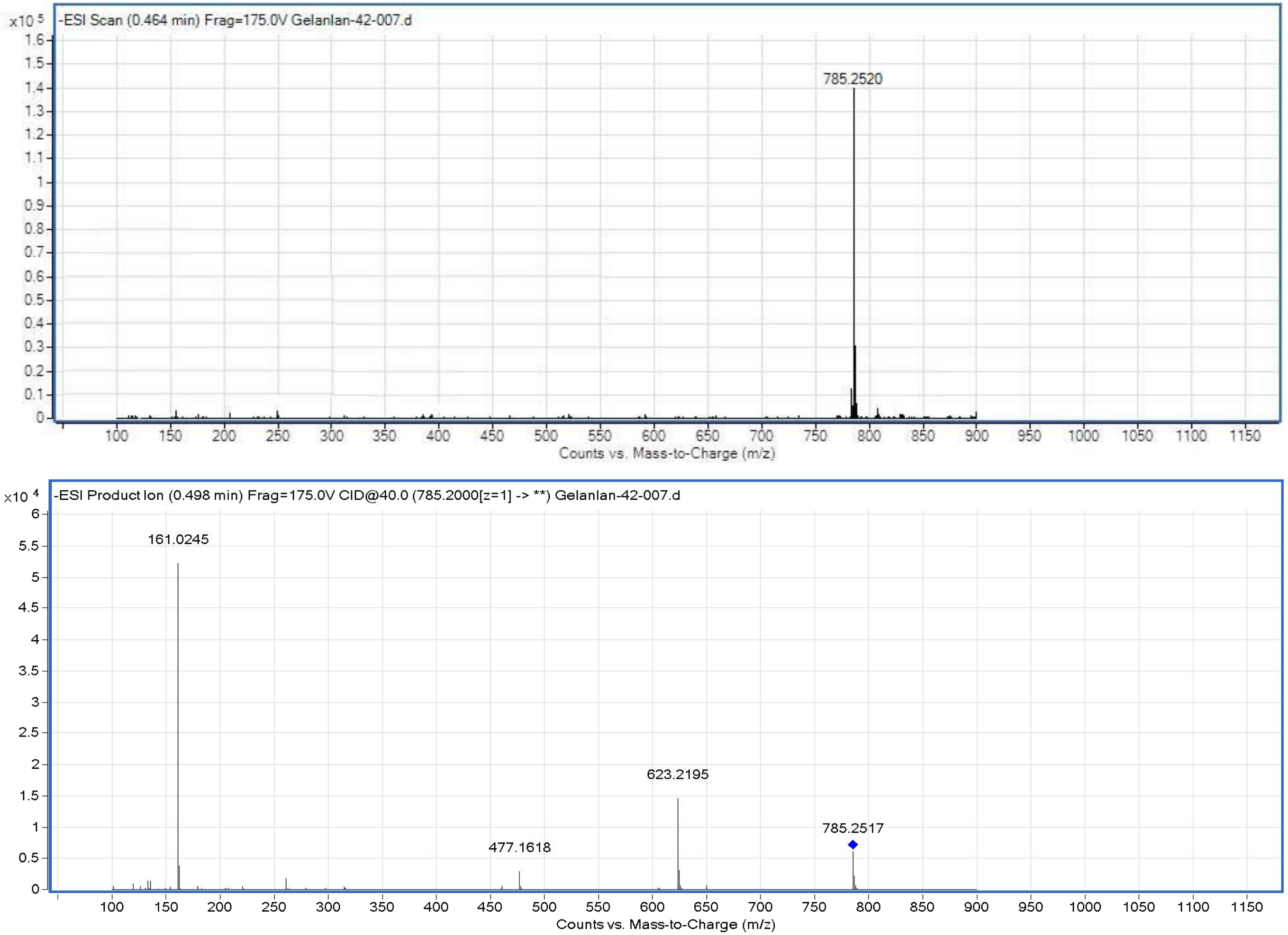

HR- ESI-MS f compound 8

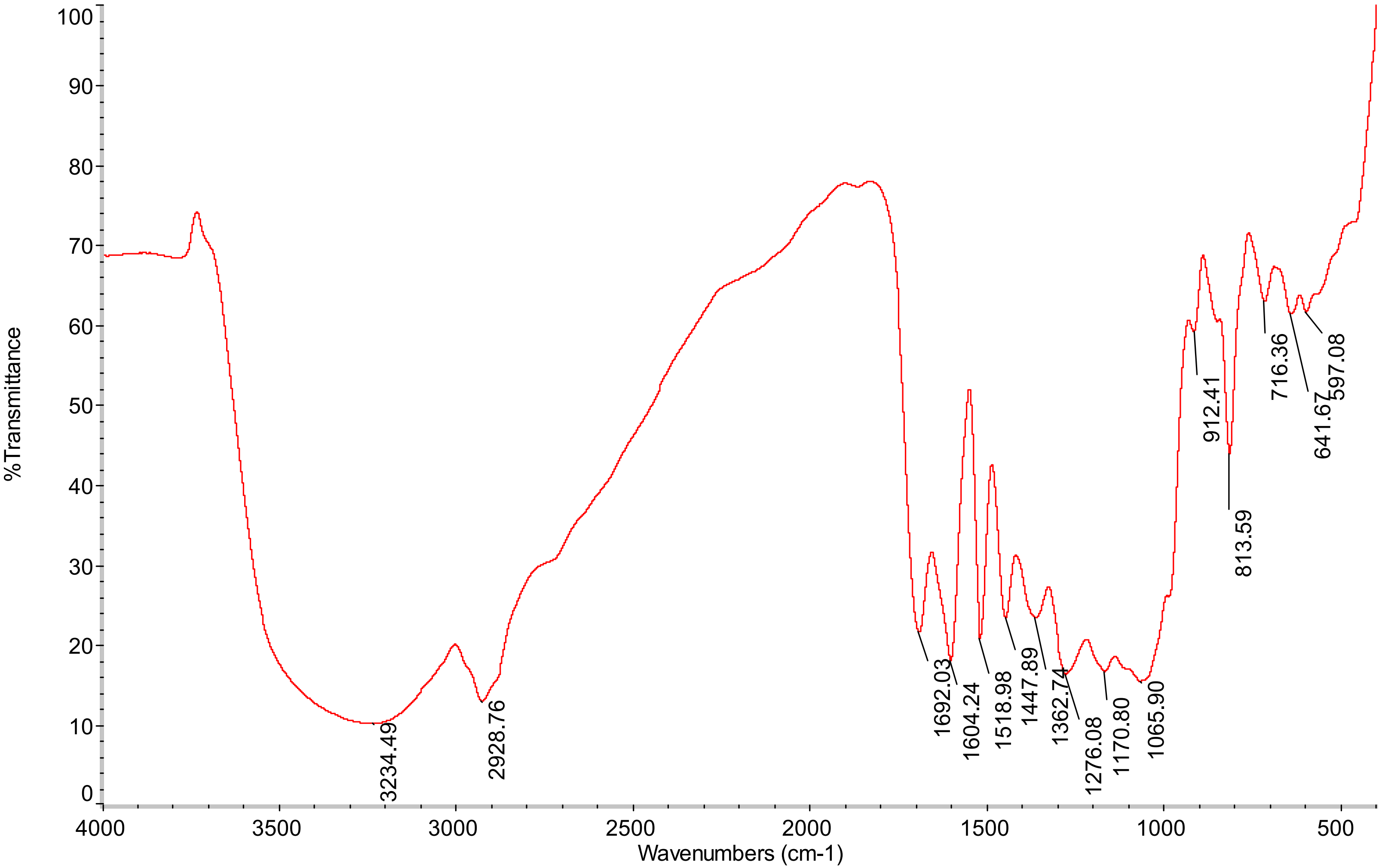

IR spectrum of compound 8

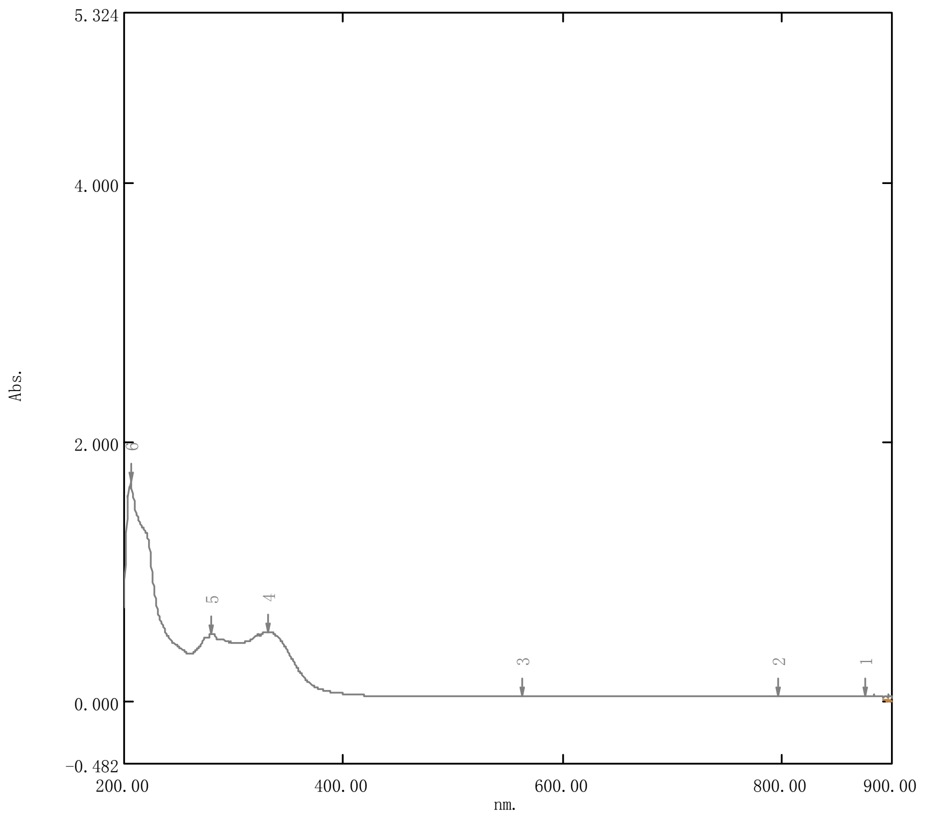

UV scan of compound 8

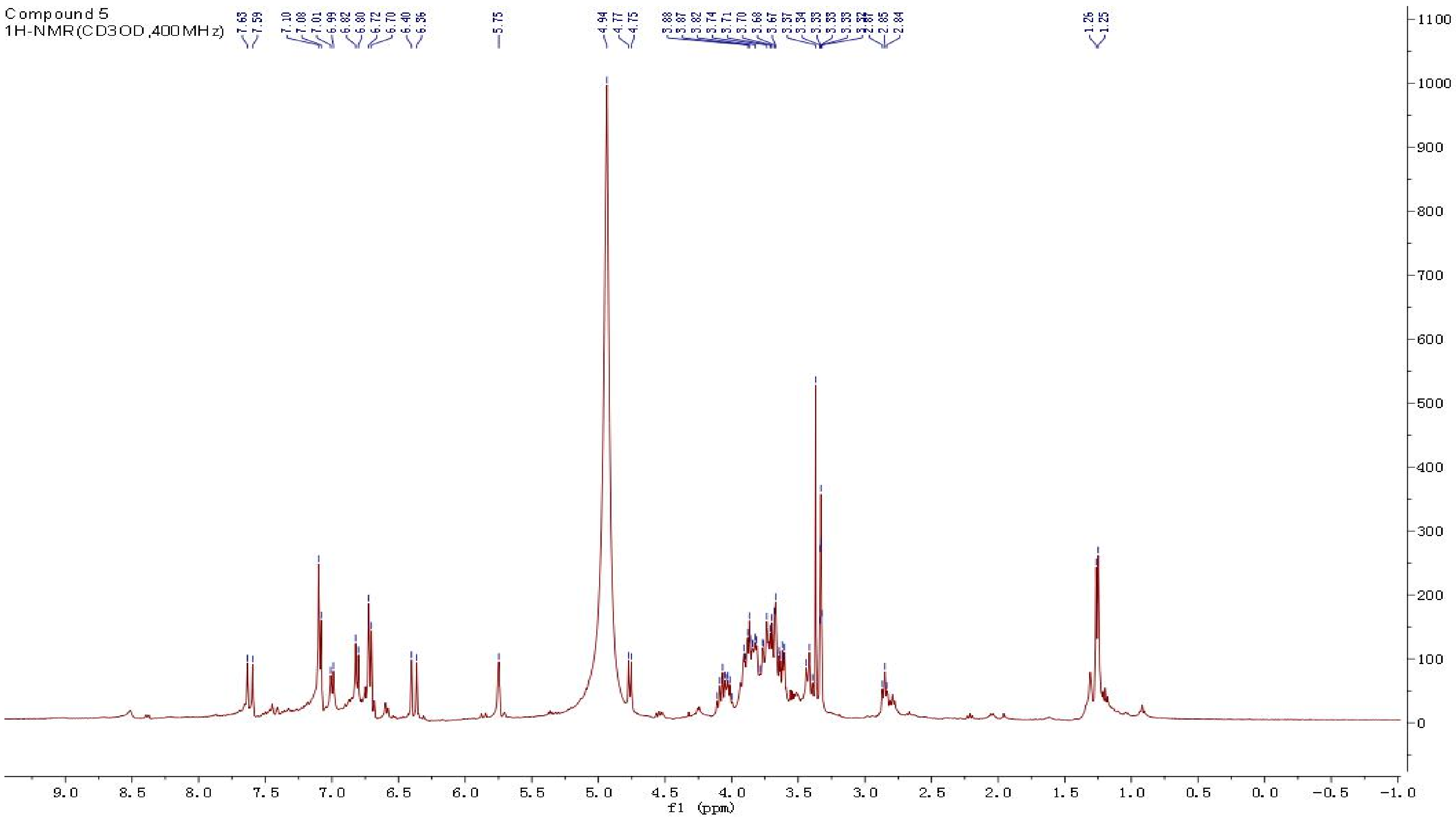

^{1}H-NMR spectrum of compound 5

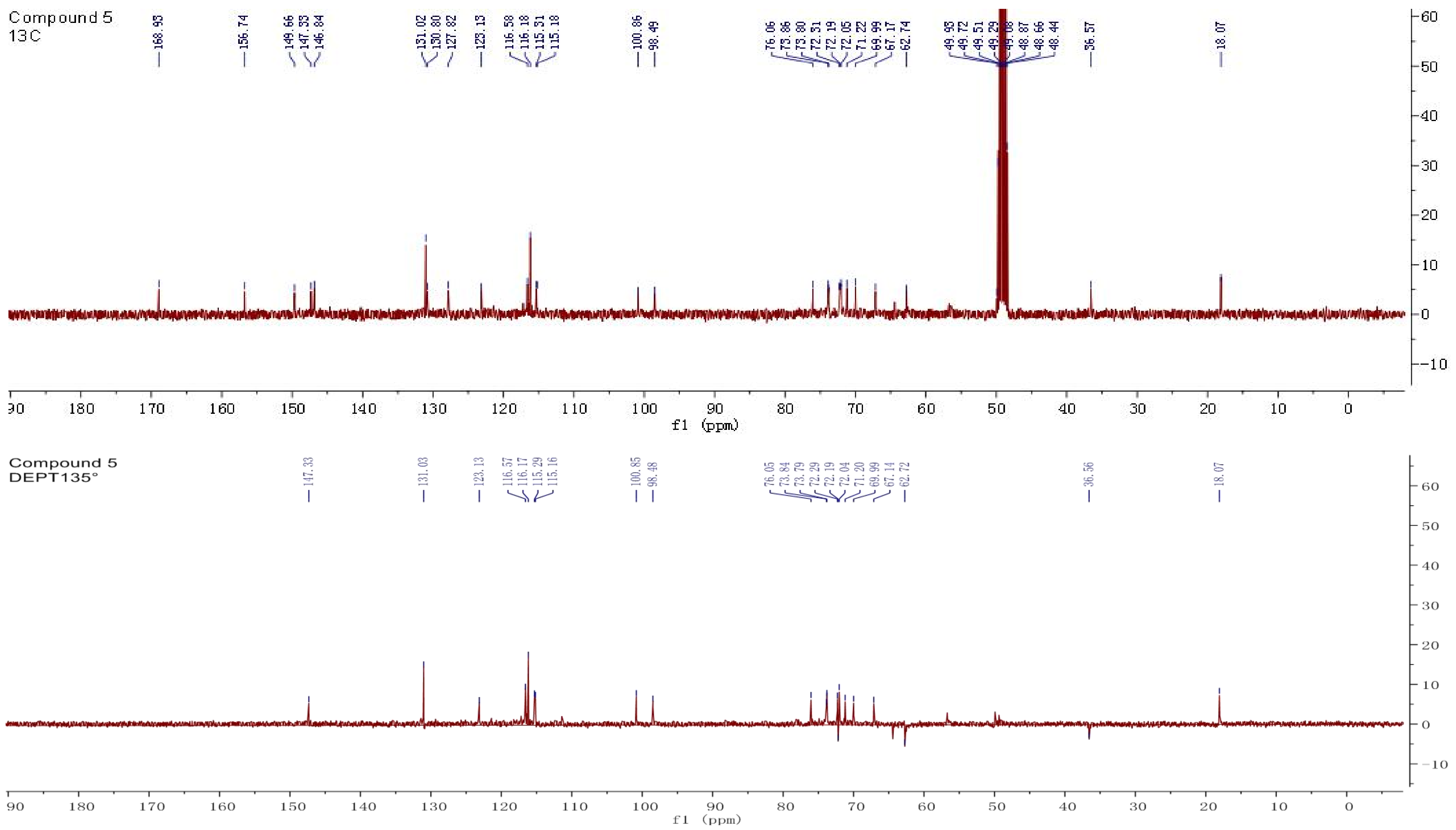

^{13}C-NMR spectrum of compound 5

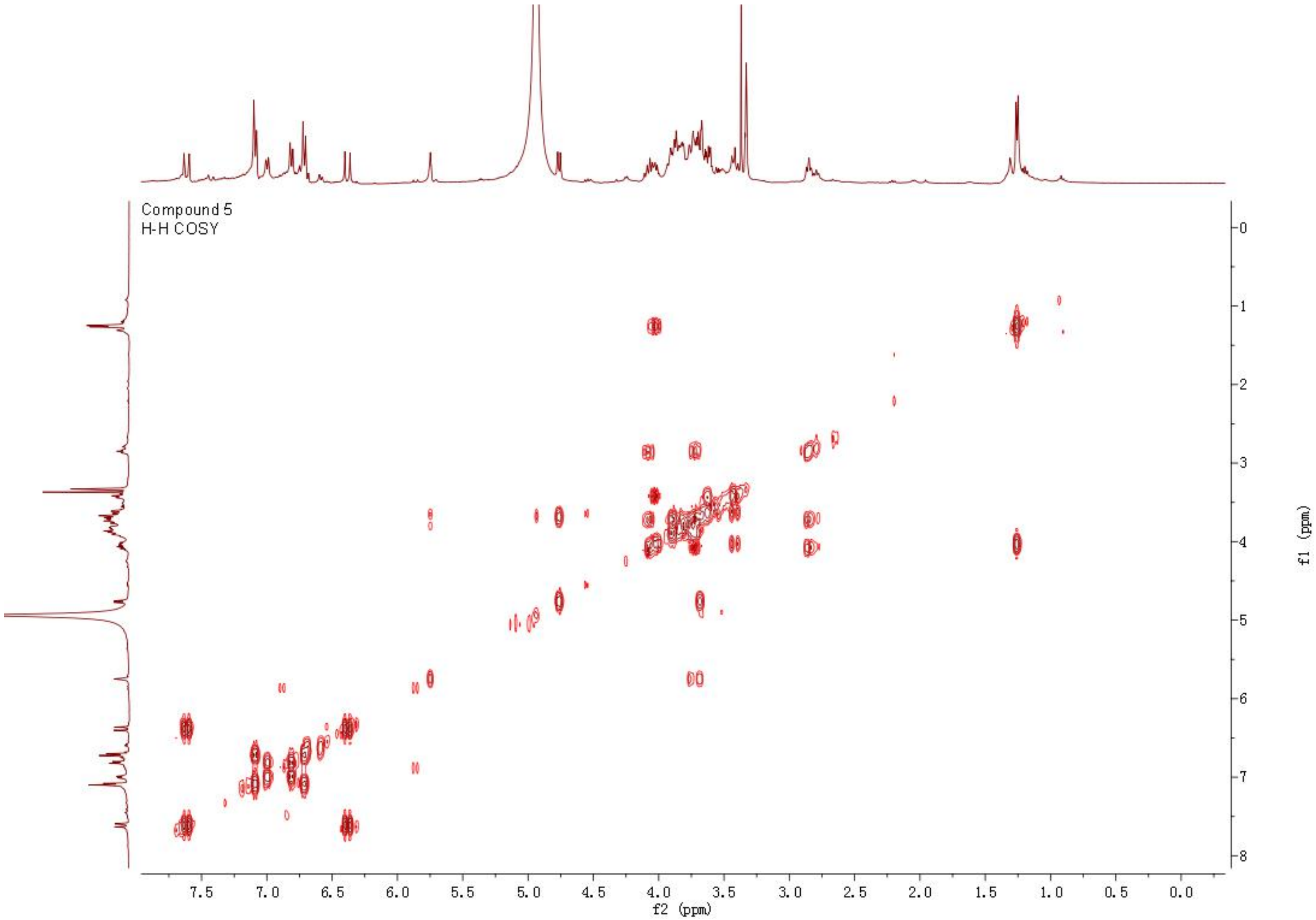

H-H COSY spectrum of compound 5

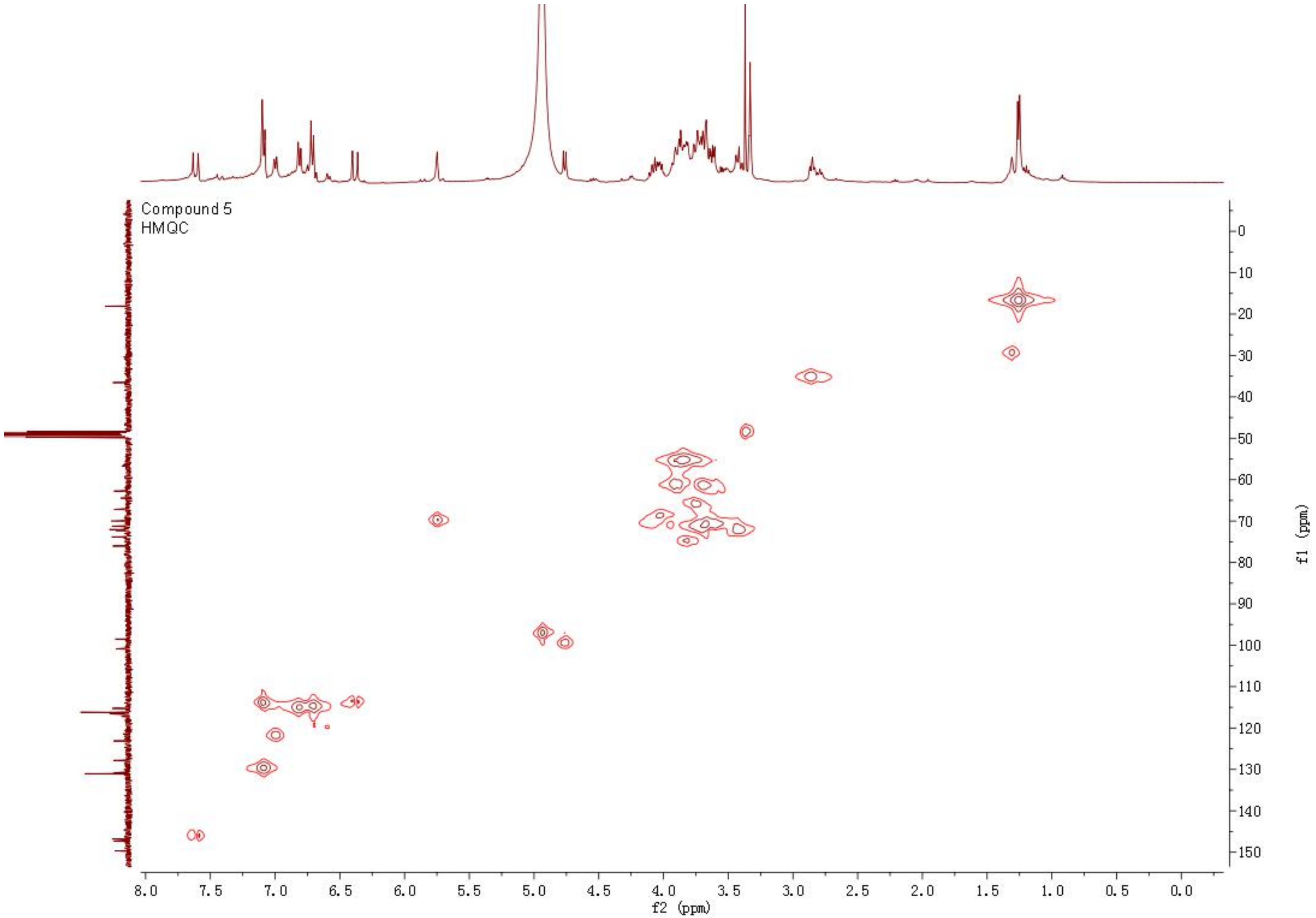

HMQC spectrum of compound 5

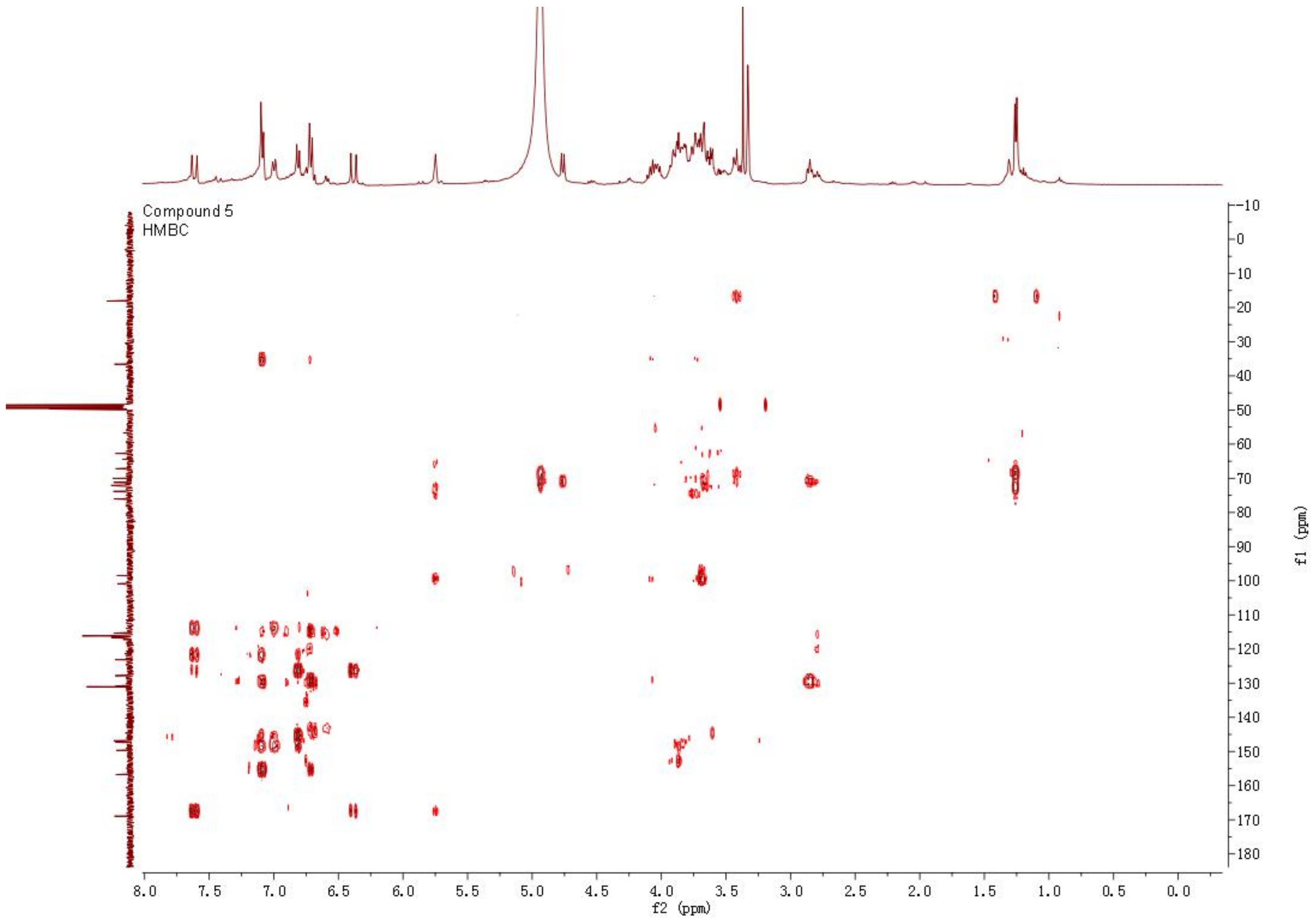

HMBC spectrum of compound 5

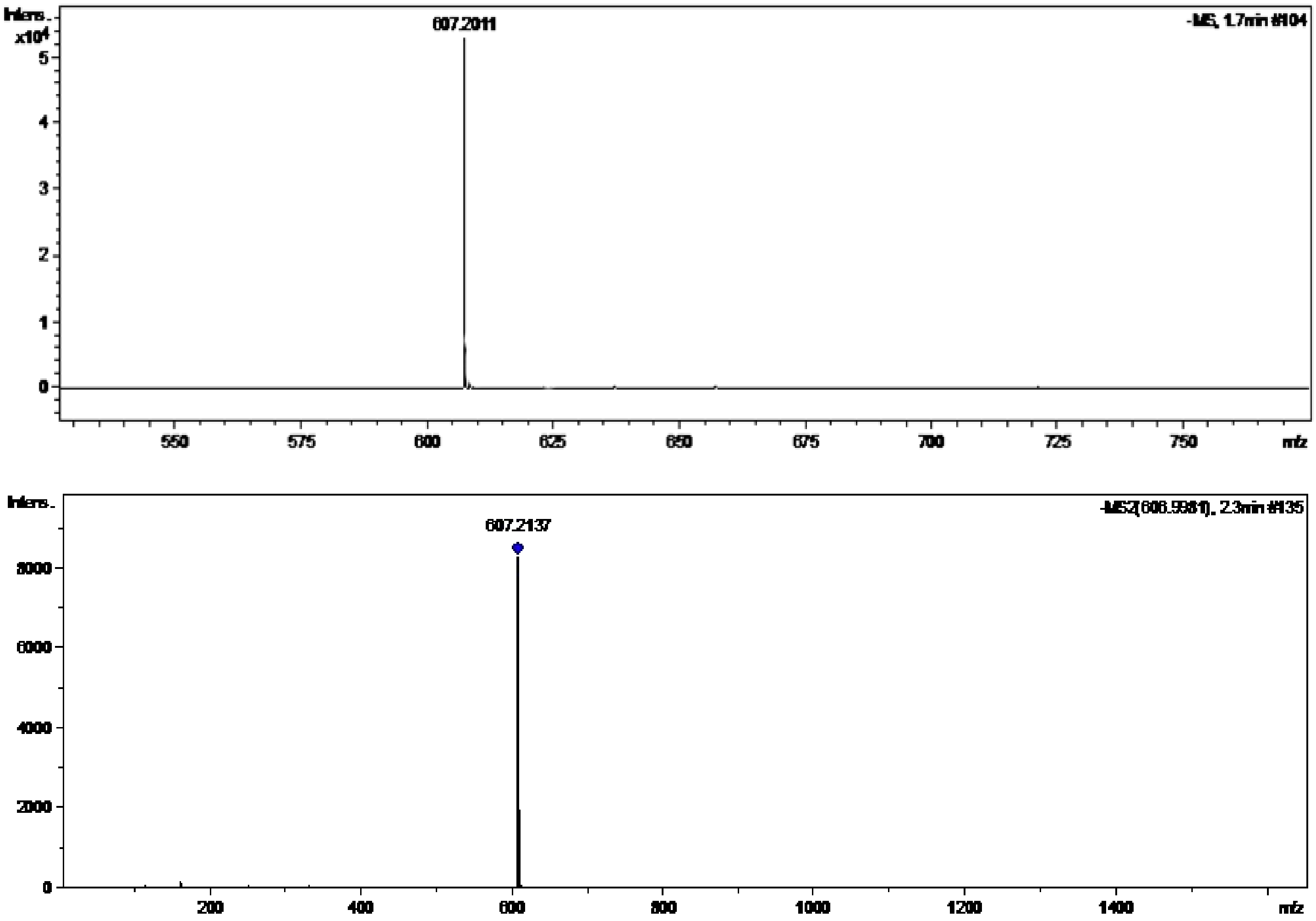

HR- ESI-MS f compound 5

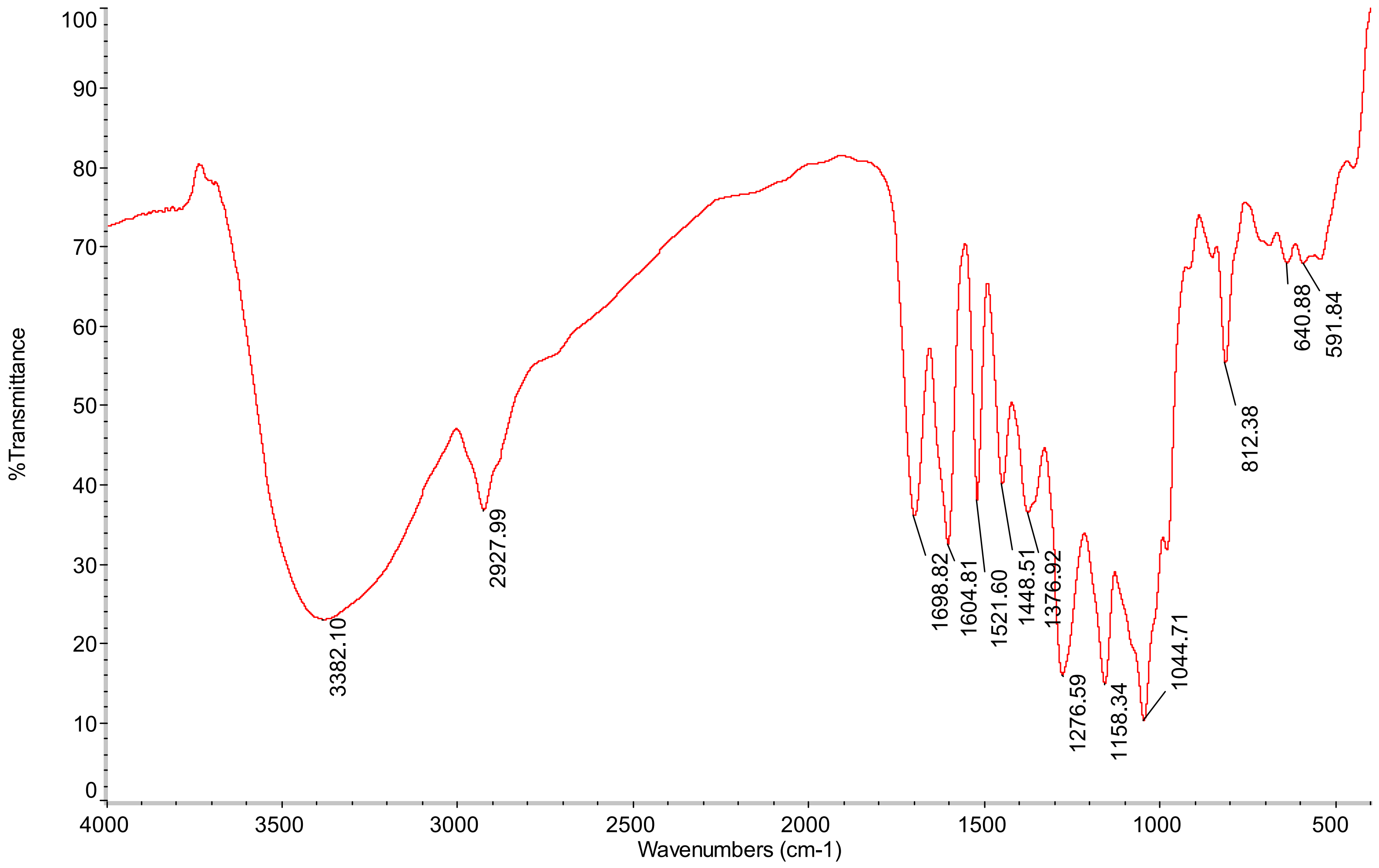

IR spectrum of compound 5

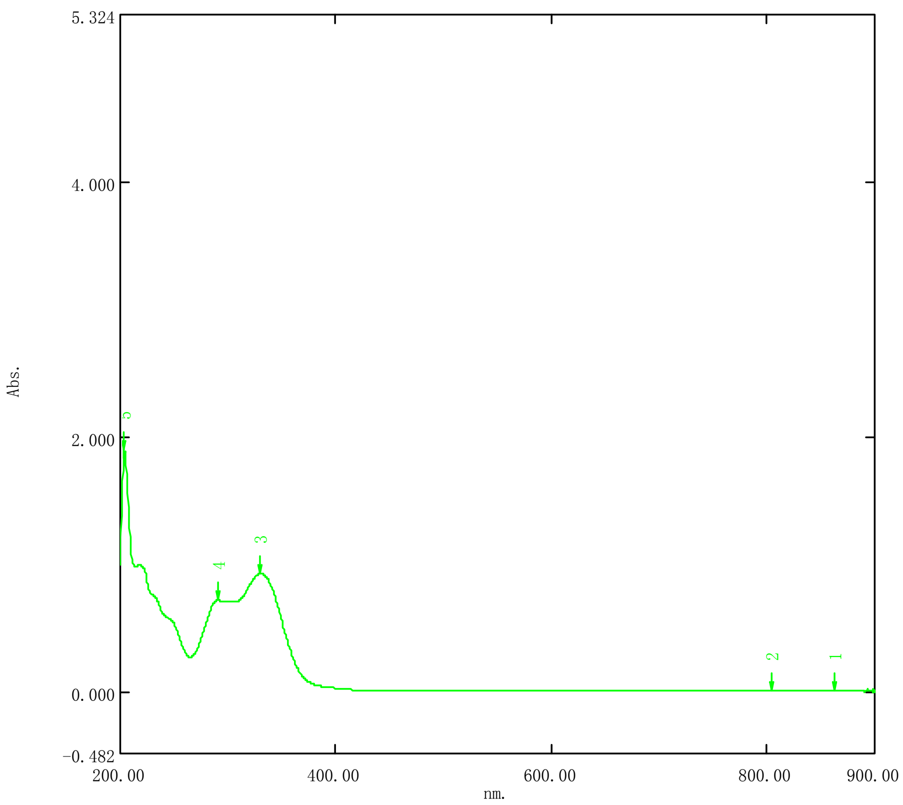

UV scan of compound 5

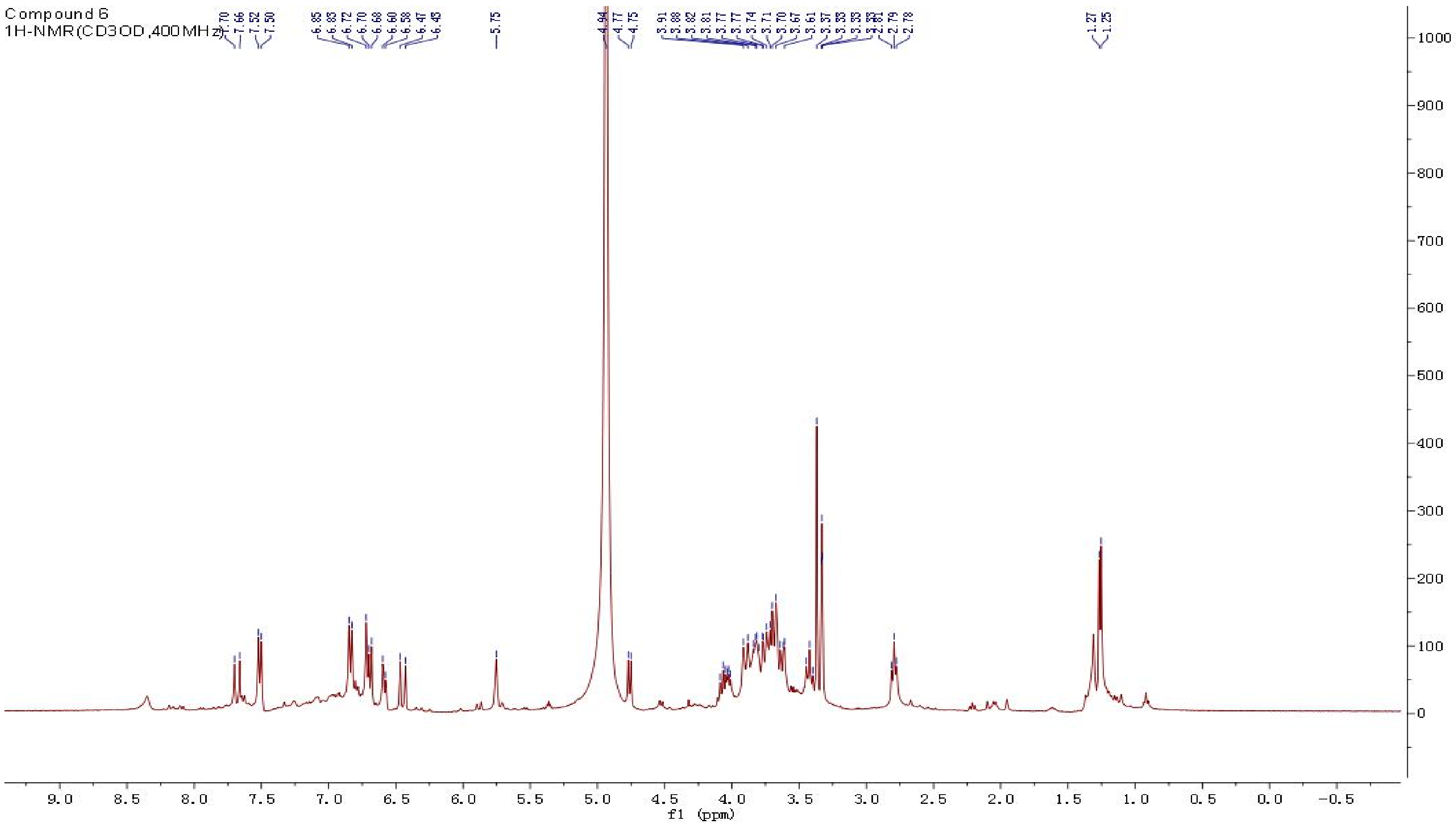

^{1}H-NMR spectrum of compound 6

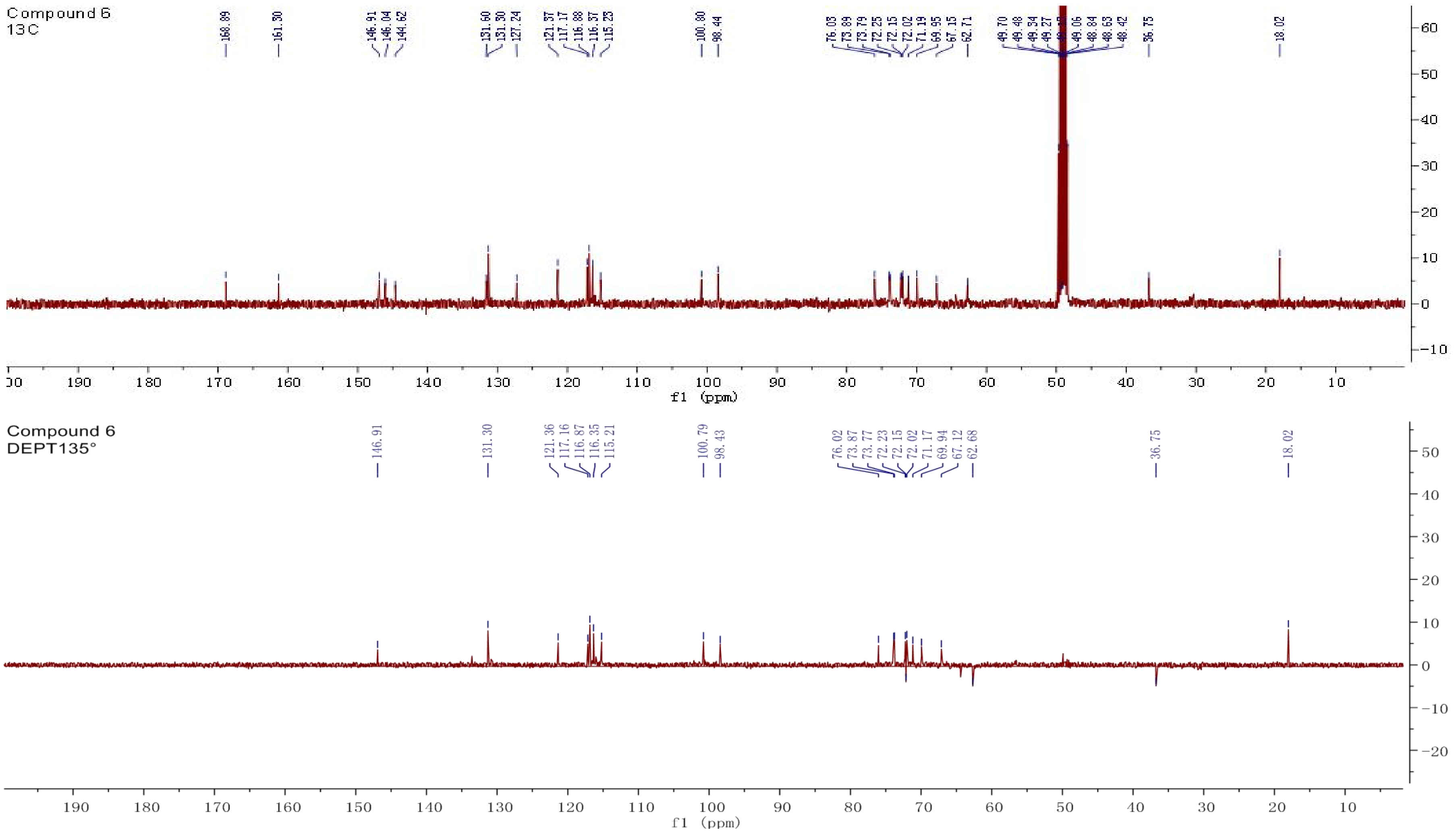

^{13}C-NMR spectrum of compound 6

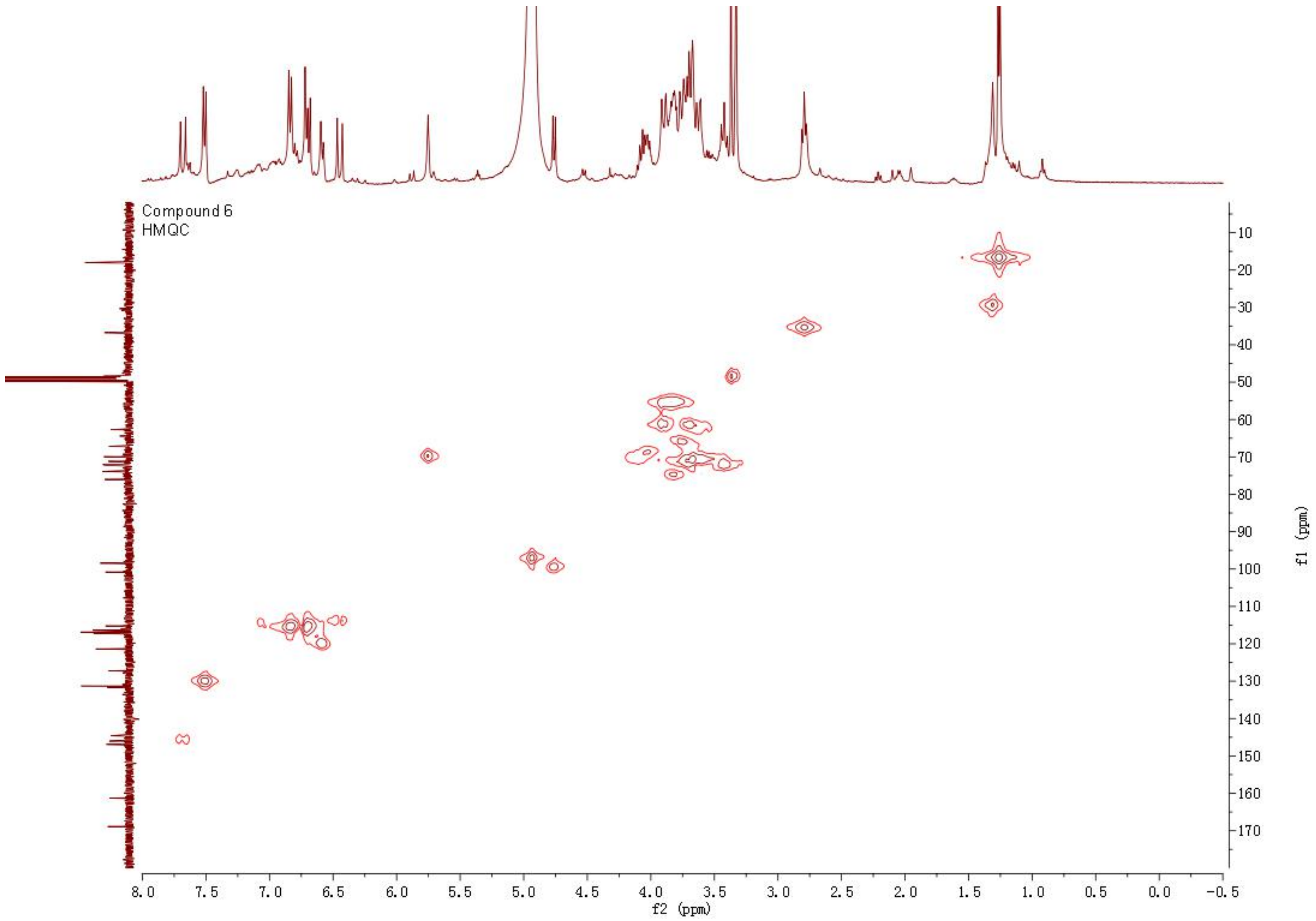

HMQC spectrum of compound 6

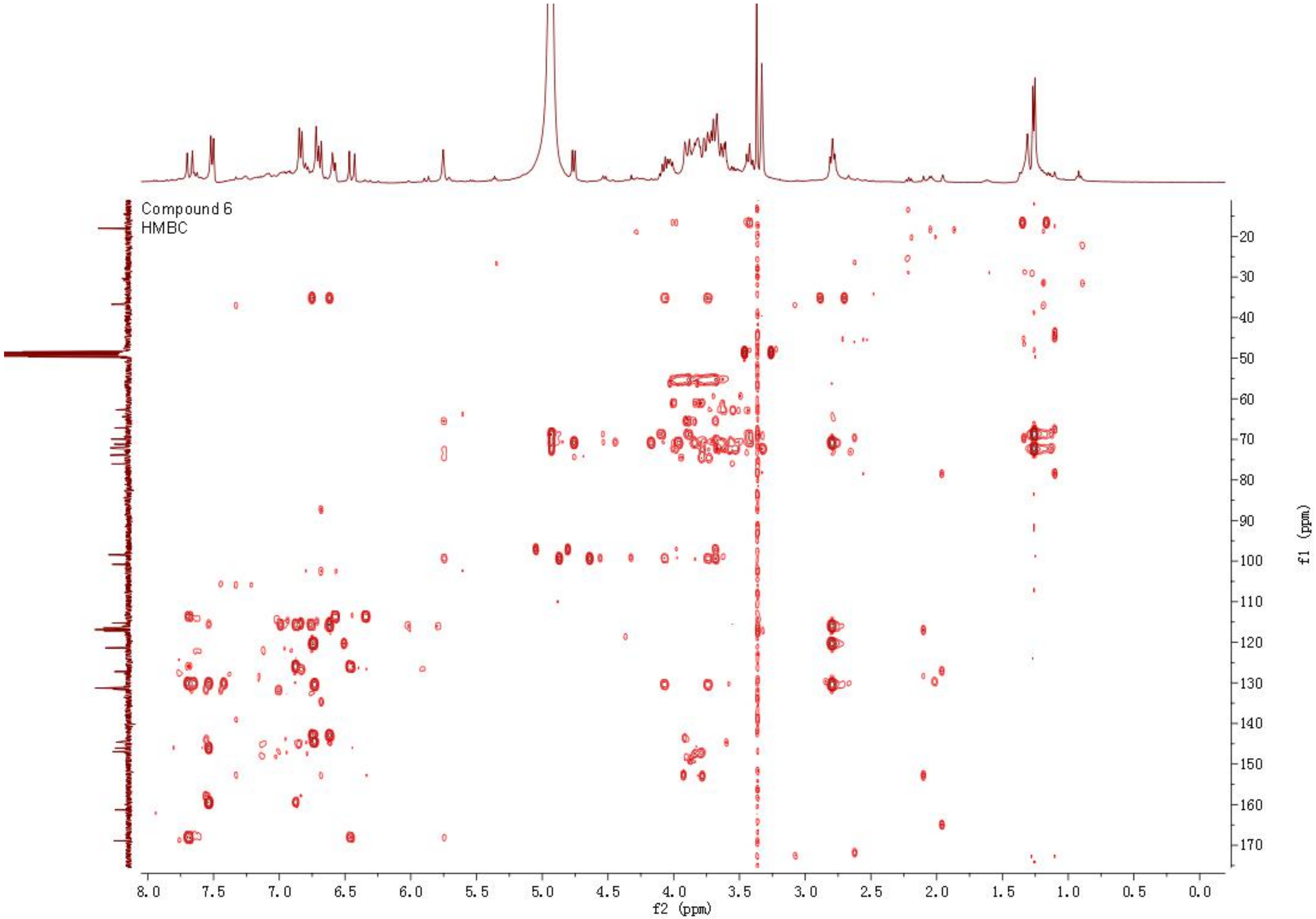

HMBC spectrum of compound 6

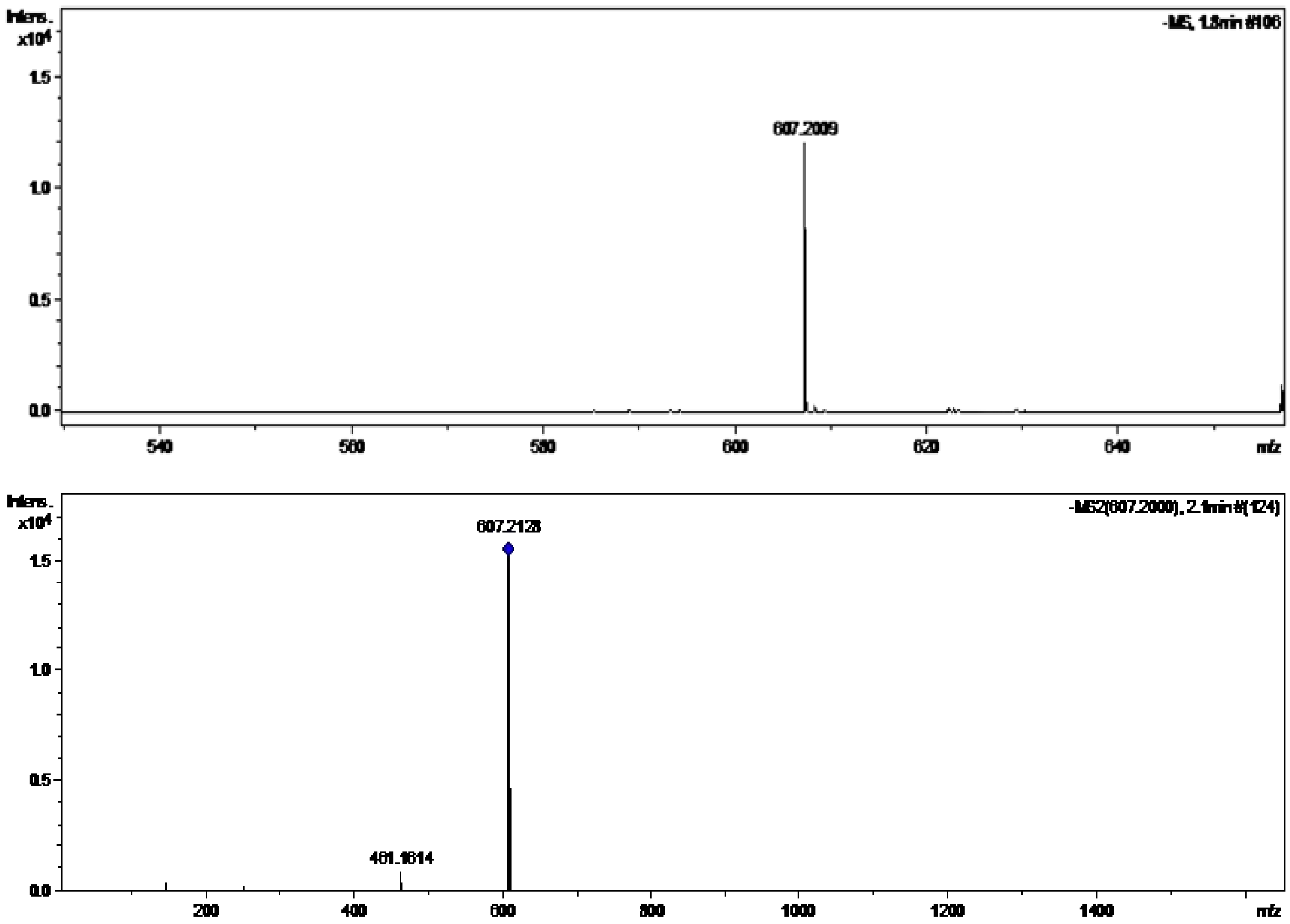

HR- ESI-MS f compound 6

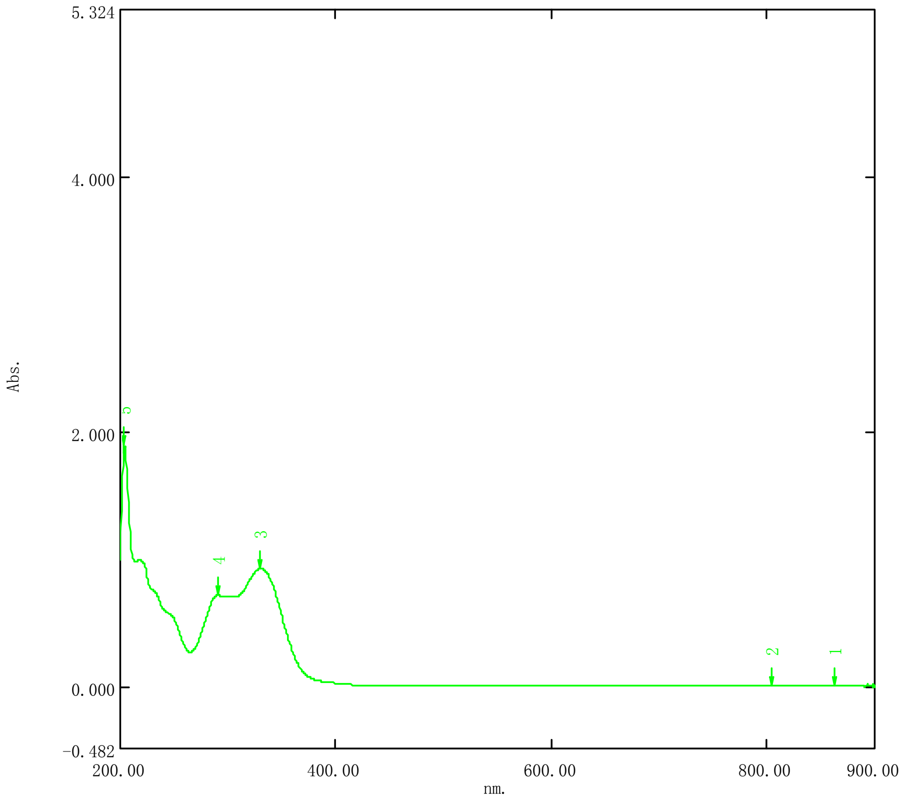

UV scan of compound 6
